# Alcohol consumption and MRI markers of brain structure and function: Cohort study of 25,378 UK Biobank participants

**DOI:** 10.1016/j.nicl.2022.103066

**Published:** 2022-05-28

**Authors:** Anya Topiwala, Klaus P. Ebmeier, Thomas Maullin-Sapey, Thomas E. Nichols

**Affiliations:** a Nuffield Department Population Health, Big Data Institute, University of Oxford, Oxford OX3 7LF, UK; b Department of Psychiatry, University of Oxford, Warneford Hospital, Oxford OX3 7JX, UK; c Nuffield Department Population Health, Big Data Institute, University of Oxford, Oxford OX3 7LF, UK; d Wellcome Centre for Integrative Neuroimaging, FMRIB, Nuffield Department of Clinical Neurosciences, University of Oxford, Oxford OX3 9DU, UK

**Keywords:** Alcohol, MRI, Cognition

## Abstract

Moderate alcohol consumption is widespread but its impact on brain structure and function is contentious. The relationship between alcohol intake and structural and functional neuroimaging indices, the threshold intake for associations, and whether population subgroups are at higher risk of alcohol-related brain harm remain unclear. 25,378 UK Biobank participants (mean age 54.9 ± 7.4 years, 12,254 female) underwent multi-modal MRI 9.6 ± 1.1 years after study baseline. Alcohol use was self-reported at baseline (2006–10). T1-weighted, diffusion weighted and resting state images were examined. Lower total grey matter volumes were observed in those drinking as little as 7–14 units (56–112 g) weekly. Higher alcohol consumption was associated with multiple markers of white matter microstructure, including lower fractional anisotropy, higher mean and radial diffusivity in a spatially distributed pattern across the brain. Associations between functional connectivity and alcohol intake were observed in the default mode, central executive, attention, salience and visual resting state networks. Relationships between total grey matter and alcohol were stronger than other modifiable factors, including blood pressure and smoking, and robust to unobserved confounding. Frequent binging, higher blood pressure and BMI steepened the negative association between alcohol and total grey matter volume. In this large observational cohort study, alcohol consumption was associated with multiple structural and functional MRI markers in mid- to late-life.

## Highlights

• Alcohol intake is associated with smaller grey matter volumes across the brain.
• It is also associated with lower FA and increased functional connectivity.
• Binge drinking steepens association between alcohol and total grey matter volume.
• Findings suggest even 7–14 units of alcohol weekly may be associated with brain differences.

## 1 Introduction

Moderate alcohol consumption is very common yet conflicting associations with late-life brain and cognitive outcomes have been reported (Visontay et al., 2021). Without disease-modifying treatments for neurodegenerative disease, there is a necessary focus on modifiable risk factors such as alcohol. Even small adverse effects of moderate drinking on the brain may have substantial public health relevance given the widespread exposure to alcohol. Here we assess relationships between alcohol consumption and neuroimaging indices. Structural MRI measures can serve as biomarkers before cognitive decline in people later developing dementia (Mcconathy and Sheline, 2015). Until the establishment of UK Biobank, costs have prohibited collection of imaging sample sizes necessary to examine the impact of drinking at such low levels.

Whilst chronic heavy alcohol intake is known to associate with reduced brain volume (Pfefferbaum et al., 1992, Mackey et al., 2019), the impact of ‘moderate’ consumption (defined variably from < 14 (Kim et al., 2020) to < 25 units (Paul et al., 2008) weekly) has been contentious (Anstey et al., 2009, Sabia et al., 2018). This is reflected in alcohol guidelines which currently neglect the brain, relying solely on cardiovascular and cancer research (Care DOHaS, 2016). Previous work has reported associations with hippocampal atrophy in males drinking just 14–21 units weekly (Topiwala et al., 2017). Two UKB studies of alcohol consumption and structural neuroimaging have reported associations between alcohol consumption, at even lower levels between 7 and 14 units weekly, and grey and white matter measures (Evangelou et al., 2021, Daviet et al., 2022). The UK Biobank has high statistical power but also higher sensitivity to confound effects (Alfaro-Almagro et al., 2021). Thus residual confounding, which could cause spurious correlations, is a concern. Earlier analyses have controlled for only a limited number of (particularly image-related) confounders. Studies to date have also failed to distinguish between never and ex-drinkers, raising the possibility that ‘sick quitters’ could be influencing results (Marmot and Brunner, 1991). Whether low levels of alcohol consumption relate to the brain’s functional architecture has not been well studied. Functional connectivity of the brain, meaning synchrony of activity between regions, changes in dementia and has been observed in small studies of alcohol dependent individuals but not examined in large samples of non-dependent drinkers (Zhu et al., 2017). What is also unclear is whether certain clinical or demographic factors increase the risk of alcohol-related brain harm. This has public health relevance in terms of focusing interventions on those at greatest need (Paper, 2020). Understanding how medical comorbidities, such as hypertension and obesity, interact with alcohol use is unknown (Qizilbash et al., 2015). ApoE4 genotype is a well-established risk factor for Alzheimer’s disease (Belloy et al., 2019). It has been implicated in brain changes in later life, including in the hippocampus (Honea et al., 2009), a structure previously associated with alcohol consumption (Topiwala et al., 2017). Whether ApoE4 modifies effects of alcohol on the brain is unclear. There are a few substantiated claims that red wine has beneficial effects (Pignatelli et al., 2006). Conversely it is thought that certain drinking patterns, such as binging, may worsen the brain impact (Hunt, 1993).

To address these unanswered issues, we investigated alcohol consumption and brain measures in UKB. We had four main research questions:

1) Are previous associations between alcohol consumption and structural neuroimaging indices reproducible in voxel-wise analyses, and robust to observed confounding?
2) Does alcohol consumption associate with functional connectivity in the brain?
3) Do the following factors increase risk of alcohol-related brain effects: ApoE4, hypertension, high body mass index, beer and spirit consumption, and binge drinking?
4) How robust are associations between alcohol and MRI markers to unobserved confounding?

An exploratory objective was to investigate the functional significance, in terms of cognitive performance, of any alcohol-brain associations.

## 2 Methods

### 2.1 Study population

UKB comprises >40,000 subjects imaged among ∼500,000 of the core study (40–69 years at recruitment, 2006–10). Participants were scanned at three centers with identical Siemens Skyra 3T scanners using a standard 32-channel head coil (Smith 2020). Imaging was performed 9.60 ± 1.10 years after study baseline (2006–10). Subjects with at least one brain MRI by 28.1.21 (n = 43,572) were included (SFigure 1). Exclusions were due to missing or insufficient quality data for analyses. Subjects who self-reported as “drinkers” but then reported 0 units weekly (n = 3760) were excluded from the analyses to avoid misclassification. Those who reported being current non-drinkers but reported any frequency of binge drinking (n = 22), and lowest quartile drinkers who reported daily binging (n = 5), were assumed to have missing data and were excluded from the analysis.

### 2.2 Alcohol consumption

At baseline participants reported their average weekly or monthly alcohol intake in number of glasses. Guidance was given about glass numbers in the normal bottle. Glasses were converted to UK units and grams (see Supplementary Methods). Non-drinkers were subdivided into former and never drinkers. Drinkers were divided into quintiles of weekly alcohol intake (whole sample) for categorical analysis. Beverage consumed and frequency of binge drinking (>six units in one episode) was sought.

### 2.3 Health-related data

Variables of interest (measured at baseline unless otherwise stated) were included as potential confounders because of associations with neuroimaging measures (Maillard et al., 2012, Herrmann et al., 2019) (for more detailed information on tests and procedures see: https://biobank.ndph.ox.ac.uk/ukb/field.cgi?id=6138 and https://www.gov.uk/what-different-qualification-levels-mean/list-of-qualification-levels): age at scanning and sex, smoking status (reported in categories: never/previous/current). Educational qualifications, from high to low, were reported as: college or university degree, A levels or equivalent, O levels or equivalent, CSEs (Certificate of Secondary Education) or equivalent, NVQ (National Vocational Qualification) or equivalent, other professional qualifications, or none (lowest level used as reference). Systolic (SBP) and diastolic (DBP) blood pressure were automated measurements. Body mass index (BMI) was calculated from measured height and weight. Townsend Deprivation Index was used as a continuous measure of deprivation based on census information. Weekly exercise expenditure was measured in Metabolic Equivalent of Task (MET) summed minutes of moderate or vigorous activity. Diabetes mellitus diagnoses were generated by a UKB algorithm using self-report, hospital care records, and death certificates. Subtypes of diabetes mellitus (insulin-dependent, noninsulin-dependent, unspecified were combined to generate a binary diabetes mellitus (present/absent) variable. For identification of depression and alcohol dependence cases, linked Hospital Episode Statistics (summary diagnoses) were used. These represent distinct diagnosis codes recorded across all of participants’ hospital inpatient records. Depression cases were defined using ICD 9 & 10 codes for single or recurrent episodes of at least moderate severity (see Supplementary Table for codes). Alcohol dependence cases were also defined using ICD 9 & 10 codes (see Supplementary Table for codes). Primary care records were not used as only half the UKB sample has linked records thus far.

### 2.4 Biological data

Total cholesterol and high-density lipoprotein (HDL) were measured from a blood sample at baseline. The number of copies of the ε4 allele of the apolipoprotein E gene (APoE4) were derived from v3 imputed ApoE genotype data (single nucleotide polymorphisms: rs429358 & rs7412) using qctool (version 2.0.7).

### 2.5 Cognitive performance data

Cognitive test data at imaging visit were: trail-making test (durations, reflecting executive function; numerical – ‘TMTA’; alpha-numeric – ‘TMTB’), tower rearranging (number attempted, reflecting executive function, digit span (maximum digits recalled, reflecting working memory), fluid intelligence (sum of correct answers), prospective memory (incorrect or correct on 1st/2nd attempt), pairs matching (number correctly associated, reflects visual memory), matrix pattern completion (duration spent answering each puzzle, reflects processing speed) and reaction time (mean time to correctly identify matches in a task based on the “Snap” card-game) (Fawns-Ritchie et al., 2020).

### 2.6 MRI processing

For details on MRI sequence parameters see Supplementary Methods. T1, DTI and rsfMRI images were used in this analysis. Details on UKB preprocessing and quality control pipelines can be found at: https://biobank.ctsu.ox.ac.uk/crystal/crystal/docs/brain_mri.pdf, accessed on 18/05/22.

#### 2.6.1 Structural T1-weighted images

T1 structural images were gradient distortion corrected and registered linearly and non-linearly (using FMRIB’s Linear Registration Tool, FLIRT (Jenkinson and Smith, 2001) and FMRIB’s Nonlinear Image Registration Tool, FNIRT (Andersson et al., 2007) to MNI152 “nonlinear 6th generation” standard-space. Brain extraction (using Brain Extraction Tool, BET, defacing and segmentation into tissue types (using FMRIB’s Automated Segmentation Tool, FAST (Zhang et al., 2000) were then performed. Total grey matter volume was extracted from FAST. T1 images underwent automated quality control (QC) as detailed in the UKB image processing and QC paper (Alfaro-Almagro et al., 2018). The UKB QC included checking the quality of warps, segmented tissue volumes, volumes of grey matter outside the brain mask and the amount of segmented tissue in the border of the brain mask. Volumes for subcortical structures were generated by modelling using FMRIB’s Integrated Registration and Segmentation Tool (FIRST (Patenaude et al., 2011).

The spatial distribution of associations between alcohol use and grey matter was investigated in a brain-wide hypothesis-free manner using FSL-VBM (Douaud et al., 2007) (https://fsl.fmrib.ox.ac.uk/fsl/fslwiki/FSLVBM), an optimised voxel-based morphometry (VBM) protocol (Good et al., 2001) carried out with FSL tools (Smith et al., 2004). This is an objective method to compare grey matter volume (estimated total intracranial volume adjusted) between individuals in each voxel (smallest distinguishable 3D image volume) of the structural image. Only participants with usable T1 images proceeded to the VBM analysis. After brain extraction, tissue segmentation and registration, images were averaged and flipped along the x-axis to create a left–right symmetric, study-specific grey matter template. Second, all native grey matter images were non-linearly registered to this study-specific template and “modulated” to correct for local expansion (or contraction) due to the non-linear component of the spatial transformation. The modulated grey matter images were then smoothed with an isotropic Gaussian kernel with a sigma of 2 mm. With such a large sample size we chose to perform minimal smoothing in order to achieve higher anatomical specificity of results. We created a study specific average grey matter tissue map using unsmoothed and modulated grey matter images as per standard VBM protocol. By thresholding this map (at 0.01) a grey matter mask was created. This was used as an analysis mask.

To explore the shape of alcohol-brain relationships the following image-derived phenotypes (IDPs), based on previous literature (Topiwala et al., 2017, Evangelou et al., 2021, Daviet et al., 2022), were used: total grey matter volume (from FAST), right and left hippocampus, thalamus, amygdala and putamen volumes (from FIRST). Volumes were adjusted for estimated intracranial volume (see image-related confounds below). For a post-hoc examination of an unexpected positive alcohol-volume association close to the lingual gyrus in the VBM (see results for details), volume of grey matter in lingual volume (left and right hemispheres, derived from Freesurfer generated by parcellation of the white surface using Desikan-Killiany parcellation (Klein and Tourville, 2012) was used. IDPs were standardized (z scores).

#### 2.6.2 Diffusion weighted imaging (DWI)

Diffusion images (dMRI) were corrected for eddy currents, head motion and gradient distortion. Using the tool DTIFIT (https://fsl.fmrib.ox.ac.uk/fsl/fdt) a diffusion tensor was fitted at each voxel generating fractional anisotropy (FA), tensor mode (MO), axial (L1) radial (L2, L3) and mean diffusivity (MD) maps. Tract-Based Spatial Statistics (TBSS) were used in a 4-stage process (Smith et al., 2006). Pre-processing prepared images for registration to standard space. Mean FA and the corresponding skeletonized image was created, and thresholded at 0.2. L2, L3, MD, MO skeletonized images were created, and projected onto the FA skeleton. Additionally, dMRI was fed into NODDI (Neurite Orientation Dispersion and Density Imaging (Mckee and Britton, 1998) to generate white matter microstructural parameters including intra-cellular volume fraction (icvf), isotropic water volume fraction (isovf) and orientation dispersion index (odi). Skeletonised images were averaged within a set of standard-space tract masks to generate mean values.

#### 2.6.3 Resting state functional MRI

The pipeline for rsfMRI images used MELODIC (Beckmann and Smith, 2004) which performs EPI unwarping, gradient correction unwarping, motion correction, intensity normalization and high pass temporal filtering. Artefacts were removed using independent component analysis and FMRIB’s ICA-based X-noiseifier (FIX) (Salimi-Khorshidi et al., 2014).

Resting state fMRI was used to determine large-scale brain functional connectivity. Group-averaged independent components analysis of resting state networks was carried out using a subset of subjects (4100 from first scanned participants (Alfaro-Almagro et al., 2018) using MELODIC at a dimensionality of 25. 21 of these components were used in further analyses, excluding 4 components identified as noise by the UKB processing team. The group-averaged ICA spatial maps were mapped onto each subject’s rfMRI timeseries data to derive a timeseries for each subject for each network. The standard deviations of these timeseries (‘nodes’) were used as a measure of *within* network functional connectivity (n = 21). The timeseries were also used to estimate subject-specific network matrices using FSLNets (Smith et al., 2013). Partial temporal correlations (aiming to estimate direct connection strengths better) *between* nodes’ timeseries (‘edges’, n = 210) were extracted from rsfMRI netmats.

#### 2.6.4 Image-related confounds

Standard imaging-related confounders included site. Imaging site was regressed out, instead of using mixed effects models with a random term, given there were only three sites and imbalance between group sizes. Estimated intracranial volume (=T1 scaling factor estimated when transforming from native to standard space (Smith 2020) was used as a confound in IDP analyses (STable 1). Additional image-related confounders included in sensitivity analyses were: head motion, table position, scanner acquisition parameters (site, scanner software, protocol, scan ramp, head coil). Polynomic terms for age (age^2^ and age^3^), and age × sex, age^2^ × sex interactions were included on the basis of recent demonstration of their importance in confound modeling in UKB (Alfaro-Almagro et al., 2021).

### 2.7 Statistical analyses

An overview of analyses models is given in STable 1 (see Supplementary Methods for additional details about statistical analyses). Diagnostic plots were used to check regression assumptions. For voxel-based analyses, data from single voxels in key areas of association were extracted to generate diagnostic plots. We examined differences in sociodemographic and clinical factors according to alcohol consumption using one-way ANOVA (normally distributed continuous variables), Kruskal-Wallis chi-squared for comparing medians, or χ^2^ tests of independence (categorical variables).

For VBM and TBSS, alcohol intake and covariates were demeaned (to avoid the mean signal being shared amongst many covariates) for the design matrix.

The Big Linear Model toolbox was used to perform mass univariate OLS regression (parametric inference) voxelwise (see Supplementary Methods). A p-value threshold that capped the False Discovery Rate (FDR) at 0.05 was generated using FSL’s FDR (https://fsl.fmrib.ox.ac.uk/fsl/fslwiki/FDR) and used to threshold T statistic images. As an additional sensitivity analyses in view of the large spatial extent of associations in VBM, we used a more stringent FDR threshold of 1%. Unthresholded statistical maps were uploaded to Neurovault.

Relationships between the IDPs and alcohol were assessed using linear (fixed effects) and non-linear regression models. Non-linear models comprised: 1) alcohol intake categorized into quintiles, and 2) restricted cubic splines (RCS – 5 knots, see Supplementary Methods) being applied to alcohol intake. Non-linearity was formally tested (H0: β2 = β3 = … = βk − 1 = 0) with an F-test. Associations with cognitive test performance at the time of scanning were examined. For IDP based analyses (including rsfMRI and interaction analyses), multiple comparisons were adjusted for using a conservative Bonferroni threshold. This threshold was calculated by dividing 0.05 by the number of tests performed (for example 0.05/9 in the case of subcortical ROIs).

#### 2.7.1 Sensitivity analyses

We did four sets of sensitivity analyses. First, we excluded non-drinkers from the sample and re-ran voxel-wise and IDP analyses. Second, to investigate the possibility that systolic and diastolic blood pressure and non-HDL cholesterol could be mediators rather than confounders we examined linear regression models with and without these (entered all at once). Third, we included additional image-related confounds as covariates. Fourth, we tested to what extent associations were robust to unobserved confounding (see Supplementary Methods). Partial R^2^ and robustness values were calculated using R’s sensemakr package, which estimates the necessary strength of an unobserved confounder required to fully account for alcohol effects on MRI marker (Cinelli and Hazlett, 2020).

#### 2.7.2 Subgroup and interaction analyses

Linear regression models were run amongst three separate groups of drinkers consuming solely wine, beer, or spirits. The impact of binge drinking frequency (never[reference]/less than monthly/monthly/weekly/daily) independent from total volume consumed weekly) was examined. Pre-specified subgroup analyses were performed. Interactions between alcohol (continuous) and age (continuous), sex (binary), blood pressure (continuous), BMI (continuous) and ApoE4 genotype (number of copies of ε4) were tested.

All analyses were completed in R (v3.6.0), unless otherwise stated.

## 3 Results

Participants with complete and usable imaging data included in the analysis comprised a lower proportion of females and had higher educational qualifications compared to the larger sample who attended the imaging assessment (STable 2). Most of the participants consumed alcohol (Table 1, SFigure 2). Median alcohol intake was 13.5 units (102 g) weekly. Almost half the sample (48.2%) reported drinking above current UK guidelines (14 units (112 g) weekly), but few had an ICD diagnosis of alcohol dependence in linked HES records (n = 31). Non-drinking groups comprised more females, lower rates of smoking, higher material deprivation, and fewer educational qualifications. Current drinkers had higher blood pressure and HDL levels but lower total cholesterol and BMI. Frequent binge drinking was associated with younger age, male sex, more educational qualifications, higher material deprivation, and smoking, independent of total alcohol consumed (STable 3). Wine drinkers were most frequent (76.9%) (SFigure 3) and significantly older, better educated, with lower BMI, material deprivation, and smoking levels (STable 4).

**Table 1:** Baseline characteristics for included sample (n = 25,377 [N = 1 subject included in the VBM analysis was excluded here due absence of alcohol status data.]) by drinking status. Mean (standard deviation) values are given for normally distributed continuous variables, median (interquartile range) for non-normally distributed variables, and numbers (percentages) for categorical variables. Only selected qualification categories are presented for brevity. Group differences were calculated using one-way ANOVA for continuous variables, Kruskal-Wallis rank sum test for comparing medians, and chi-square tests for categorical variables.

Baseline characteristic	Never drinkers (N = 691)	Former drinkers (N = 617)	Current drinkers (N = 24,069)	Group differences
Age band, N(%)				
40- <50 years	206 (29.8)	171 (27.7)	6083 (25.3)	X^2^ = 16.1, df = 4,p = 0.003
50- <60 years	215 (31.1)	241 (39.1)	8892 (36.9)	
60- <70 years	200 (28.9)	153 (24.8)	6756 (28.1)	
Sex, female N(%)	457 (66.1)	320 (51.9)	11,477 (47.7)	X^2^ = 94.8, df = 2,p < 2.2 × 10^−16^

Smoking status, N(%)				
*Never*	603 (87.3)	315 (51.1)	14,119 (58.7)	X^2^ = 250.5, df = 4, p < 2.2 × 10^−16^
*Previous*	61 (8.8)	253 (41.0)	8428 (35.0)	
*Current*	27 (3.9)	49 (2.9)	1522 (6.3)	

Educational qualifications, N(%)				
*None*	66 (9.6)	54 (8.8)	1264 (5.3)	X^2^ = 55.9, df = 12, p = 1.24 × 10^−7^
*A level*	86 (12.5)	87 (14.1)	3145 (13.1)	
*Degree*	301 (43.6)	263 (42.6)	11,813 (49.1)	
Systolic blood pressure, mmHg	134.28 (18.8)	134.02 (17.8)	137.3 (18.7)	F(2,25374) = 17.4, p = 2.9 × 10^−8^
Diastolic blood pressure, mmHg	79.7 (10.8)	79.7 (10.2)	81.7 (10.5)	F(2,25374) = 21.5, p = 4.9 × 10^−10^
Body Mass Index, kg/m^2^	26.7 (4.7)	27.0 (4.7)	26.5 (4.0)	F(2,25374) = 5.7, p = 0.003
Total cholesterol, mmol/L	5.6 (1.2)	5.54 (1.2)	5.73 (1.1)	F(2,25374) = 13.1, p = 2.2 × 10^−6^
Non-high Density cholesterol, mmol/L	4.2 (1.0)	4.2 (1.1)	4.3(1.1)	F(2,25374) = 0.001, p = 1.0
Diabetes Mellitus, N(%)	57 (8.3)	40 (6.5)	1139 (4.7)	X^2^ = 21.5, df = 2, p = 2.2 × 10^−5^
Townsend Deprivation Index^2 4^	−1.4 (3.0)	−0.8 (3.2)	−2.0 (2.6)	F(2,25374) = 72.7, p = 2.0 × 10^−16^
Exercise, Metabolic Equivalent Task minutes weekly	90 (1 1 8)	105(1 2 0)	100 (1 0 0)	X^2^ = 7.1, df = 2,p = 0.03

### 3.1 Alcohol and structural T1-weighted images

**Fig. 1:**
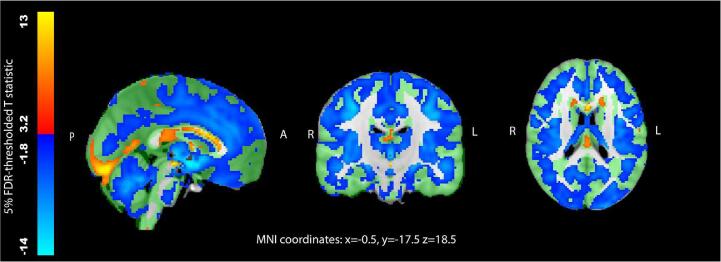
Associations between weekly alcohol intake and grey matter volume generated using voxel-based morphometry. T statistics are thresholded at 5% False Discovery Rate (0.028 threshold on uncorrected p values for negative associations and 0.001 uncorrected p values for positive associations). N = 25,378. Study specific grey matter mask is shown in green. Adjusted for: age, sex, age^2^, age^3^, age × sex, age^2^ × sex, imaging site, systolic and diastolic blood pressure, total cholesterol, high-density lipoprotein, diabetes mellitus, smoking, body mass index, Metabolic Equivalent Task minutes weekly, Townsend Deprivation Index, depression, educational qualifications. Abbreviations: L – left, R – right, A – anterior, P – posterior.

Alcohol consumption was inversely associated with grey matter volume in a widespread distribution in the VBM analysis (Fig. 1). Associations were evident in the cerebellum, brainstem, frontal, parietal, occipital and temporal lobes and several subcortical structures. The strongest effects were observed in pre- and post-central gyri, supplementary motor cortex and the thalami. Although there were several significant voxels within the hippocampus, associations were not observed throughout the entire structure. Using a more stringent FDR threshold (1%) made little difference to the extent of the associations (690,096 vs. 883,960 significant voxels). A small number of voxels, many near the lingual gyrus and corpus callosum, showed positive associations with alcohol. However positive associations were not replicated in a post-hoc region-of-interest analysis of lingual volume, but rather negative associations were observed (left: beta = −1.38, 95% CI: −2.36 to −0.41, p = 0.006; right: beta = −1.76, 95% CI: −2.78 to −7.36, p = 0.0007). Structural associations were unchanged after adjustment for further MRI parameters (SFigure 4) or exclusion of non-drinkers (SFigure 5).

While the effect size was small (partial R^2^ = 0.02; SFigure 6), alcohol made a larger contribution than any modifiable risk factor tested to total grey matter volume, including smoking, BMI and blood pressure (STable 6). Adjustment for additional cardiovascular factors did not change associations between alcohol and total grey matter volume (STable 5). To relate the cross-sectional effects of age and alcohol, discounting nonlinear effects of age and age-sex interactions, we determined the effect of a 1-year difference in age on total grey matter volume was the same as a difference in 12.9 UK (∼7.3 US units, 102 g) weekly alcohol consumption (STable 7).

**Fig. 2:**
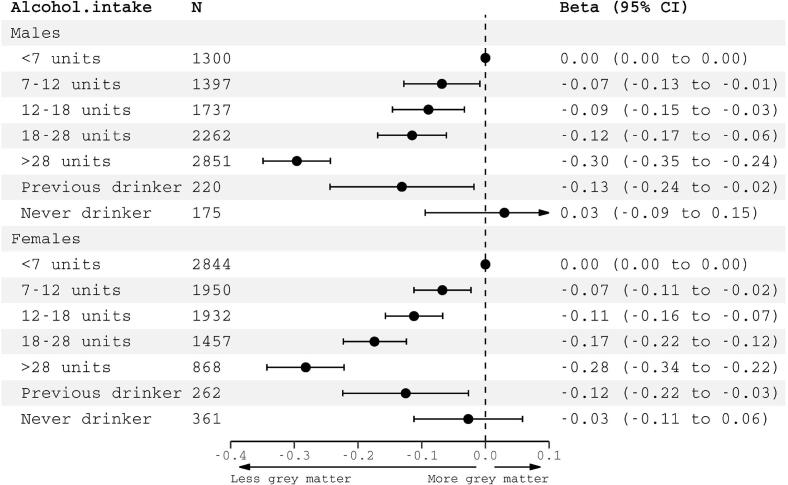
Association of total brain grey matter volume (normalized to estimated intracranial volume) with weekly alcohol intake in units. N = 22,253 participants. Alcohol categorization was based on quintiles of intake for all subjects within the sample. Beta coefficients (95% confidence intervals) reflect grey matter volume difference in standard deviations compared to the reference group of those drinking < 7 units (56 g) weekly. Models were adjusted for: age, age^2^, imaging site, systolic and diastolic blood pressure, total cholesterol, high-density lipoprotein, diabetes mellitus, smoking, body mass index, Metabolic Equivalent Task minutes weekly, Townsend Deprivation Index, depression, educational qualifications.

Those drinking >7 units (56 g) weekly (quintiles 2–5) had smaller total grey matter volumes compared to those drinking <7 units weekly (Fig. 2). Previous drinkers also had less total grey matter than the lightest drinkers, whereas never drinkers were indistinguishable from light drinkers.

The spline model did not offer significantly improved fit over a linear effect of alcohol in the model fitting total grey matter (df = 3, F = 1.6, p = 0.2; SFigure 7 – top left plot). Positive slopes between 0 and 5 alcohol units for the left putamen, right thalamus, and left and right hippocampi and amygdala were flattened upon excluding previous drinkers, possibly suggesting a “sick quitter” effect. Of the subcortical regions tested, associations between alcohol and bilateral hippocampi, putamen and thalamus survived correction for multiple comparisons. The strongest associations were found with thalamus volumes (STable 8).

### 3.2 Alcohol and diffusion-weighted images

**Fig. 3:**
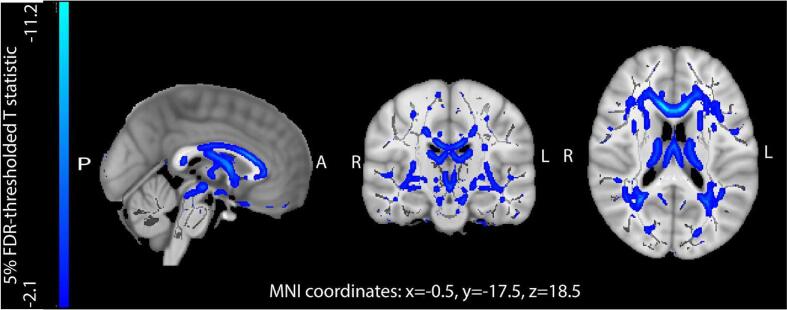
Negative associations between weekly alcohol intake and fractional anisotropy – a diffusion tensor imaging measure of white matter integrity. T statistics are thresholded at 5% False Discovery Rate (0.017 threshold on uncorrected p values). Mean fractional anisotropy skeleton shown in grey. N = 24,030. Adjusted for: age, sex, age^2^, age^3^, age × sex, age^2^ × sex, imaging site, estimated intracranial size, systolic and diastolic blood pressure, cholesterol, high-density lipoprotein, diabetes mellitus, smoking, body mass index, Metabolic Equivalent Task minutes weekly, Townsend Deprivation Index, depression, educational qualifications. Abbreviations: L – left, R – right, A – anterior, P – posterior.

Widespread negative associations were found with FA and mode, and positive associations with MD, L1, L2 and L3 across the skeleton (Fig. 3 & SFigure 10-17). Adjusting FA analyses for global mean FA reduced associations in insula, temporal and frontal tracts (SFigure 11). Associations in other regions, such as the corpus callosum and fornix appeared to be specific and not dependent on global mean FA.

### 3.3 Alcohol and resting state functional MRI

**Fig. 4:**
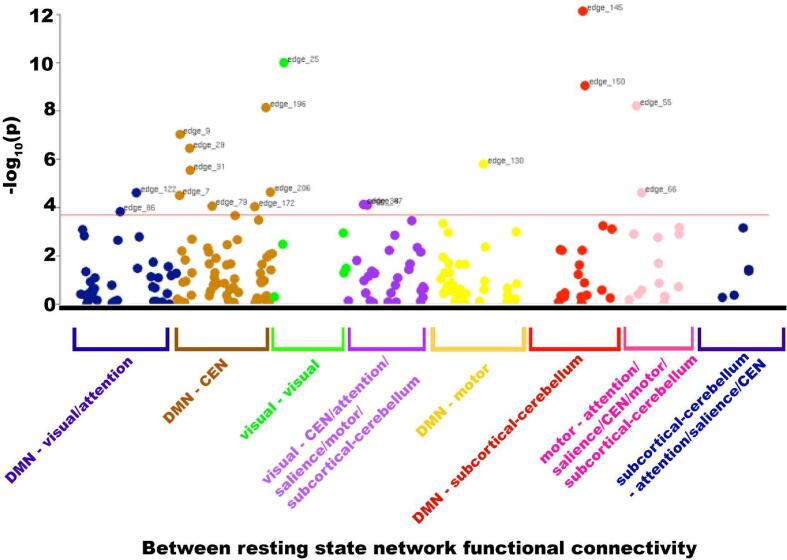
Associations between alcohol intake and between resting state network connectivity (‘edges’). Manhattan plot showing –log_10_(p) values for regression models with edge functional connectivity as the dependent variable and alcohol intake as an independent variable, adjusted for: age, sex, age*sex, age^2^, age^2^*sex, systolic and diastolic blood pressure, diabetes mellitus, smoking status, Townsend Deprivation Index, body mass index, total cholesterol, high-density lipoprotein, imaging site, head motion and Metabolic Equivalent Task minutes weekly. Bonferroni p-value threshold is shown by the red line (p < 0.0002) and edges passing this threshold are labelled. N = 17,587. Edges are represented by dots, coloured according to the resting state networks their compromising nodes reside in. Abbreviations: DMN – default mode network, CEN – central executive network.

Alcohol consumption was significantly associated with functional connectivity within seven resting-state networks (‘nodes’ 3,4,5,6,9,13,21) (Stables 11 & 12). These nodes correspond to connectivity within the default mode (nodes 5,6,9,13,21), attention (nodes 3,5), central executive (nodes 3,5,6,9,21), visual (node 4) and salience (nodes 3,13) networks. In all cases, higher alcohol intake associated with increased connectivity within the network, except for the visual network where the inverse relationship was observed (SFigure 18). Alcohol intake was additionally associated with functional connectivity strength between several resting-state networks (‘edges’) despite multiple testing correction (Fig. 4, SFigure 19 & STable 13).

### 3.4 Subgroup and interaction analyses

**Fig. 5:**
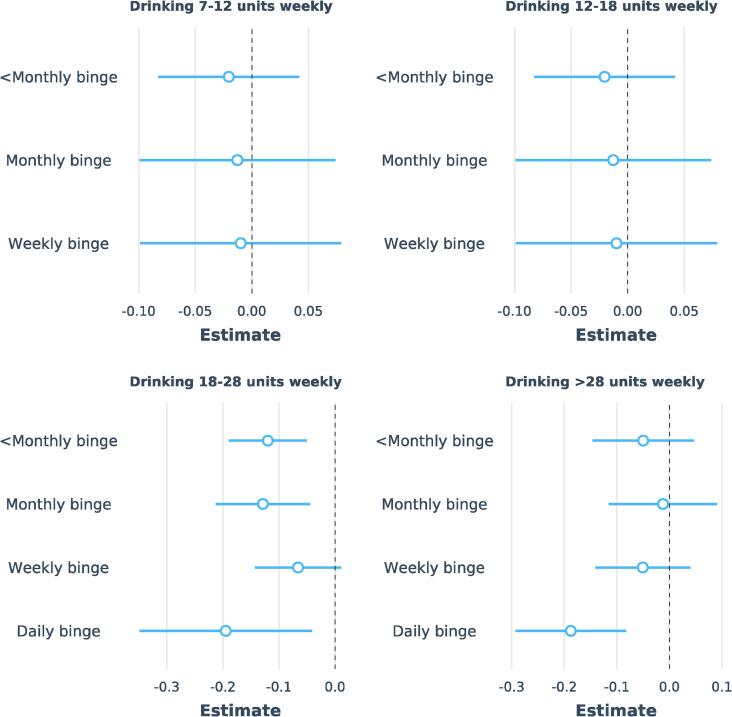
Relation between binging frequency (>6 units/48 g alcohol in one episode) and total grey matter volume (normalized to estimated intracranial volume), independent of alcohol consumption in units. N = 12,812. Points show standardized regression coefficients (estimates and their 95% confidence intervals) for binging frequency category compared to the reference category (never binging) generated from regression models with grey matter volume as the dependent variable. Results are shown separately according to subjects’ weekly alcohol intake (divided into quantiles): 1) 6.8–11.6 units (54.1–92.8 g), 2) 11.6–17.8 units (92.8–142.4 g), 3) 17.8–28.4 units (142.4–227.2 g), 4) 28.4–163 units (227.2–1304 g). N = 14,685. Regression models were adjusted for: alcohol consumption in weekly units, age, age^2^, age^3,^ sex, age × sex, age^2^ × sex, diabetes mellitus, systolic and diastolic blood pressure, body mass index, total cholesterol, high-density lipoprotein, smoking status, imaging site, Metabolic Equivalent Task minutes weekly, depression, educational qualifications, Townsend Deprivation Index.

Daily bingers had significantly lower total grey matter volume than never-bingers, even after controlling for total alcohol consumed weekly (Fig. 5). This was apparent in those drinking >18 (UK) units (∼10 US units, 144 g) weekly. Associations with total grey matter volume were not significantly different whether the weekly units were consumed as wine, beer, or spirits (see overlapping 95% CI in SFigure 22).

High blood pressure and BMI steepened the negative association between alcohol and total grey matter volume (SBP*alcohol: beta = -0.01, 95% CI = −0.02 to −0.004, p = 0.005; DBP*alcohol: beta = −0.01, 95%CI = −0.02 to −0.004, p = 0.006; BMI*alcohol: beta = −0.01, 95% CI = -0.02 to −0.002, p = 0.02) (SFigure 23). Adjustment for antihypertensive medication made no difference (STable 15). There were no significant interactions between alcohol and age, sex or ApoE4 genotype that survived correction for multiple testing (STable 16).

### 3.5 Robustness against unobserved confounding

We estimated that to nullify the effect of alcohol, an unobserved confounder would need to explain both >12% of total grey matter volume and >12% of alcohol intake variation (robustness value = 0.12). The presence of an unobserved confounder which achieves this seems implausible given examination of the strongest known confounders. For example, whilst age explains 23% of total grey matter variation, it only explains 0.1% of alcohol intake, and an unobserved confounder with such characteristics would be unable to explain the alcohol-grey matter relationship (SFigure 8). Similarly, sex and smoking each explain only 0.2% of total grey matter volume and 4% of alcohol intake variation, suggesting there is no likely unobserved confound that could account for the observed alcohol-grey matter relationship.

### 3.6 Functional relevance

Total grey matter volume was positively associated with matrix puzzle completion (beta = 0.04, 95% CI = 0.02–0.06, p < 0.001) and tower correlation (beta = 0.04, 95% CI = 0.02–0.06, p < 0.001), and negatively associated with duration of TMT A (beta = -0.05, 95% CI = −0.07 to −0.03, p < 0.001) & B (beta = −0.05, 95% CI = -0.07 to −0.03, p < 0.001) as well as reaction time (beta = −0.05, 95% CI = −0.07 to −0.03, p < 0.001) (Stable 9). Increased connectivity of several resting state networks (nodes 5, 6, 13 and 21 within CEN, attention, DMN, salience networks, STable 11) associated with performance on digit span, matrix puzzle, tower correlation and fluid intelligence after multiple testing correction (SFigure 20). Functional connectivity of node 3 was positively associated with tower correlation but inversely correlated with digit span. Controlling for alcohol consumption did not alter the associations between functional connectivity and cognitive performance, other than reducing that between digit span and connectivity of node 13 (SFigure 21).

No direct associations between alcohol and cognitive test performance were observed. There were weak interactions between alcohol and certain educational qualification categories, although none survived multiple testing correction (STable 10).

## 4 Discussion

In this large population-based neuroimaging study, alcohol was linearly and negatively associated with total grey matter volume. Higher alcohol intake was negatively associated with FA and mode, and positively associated with MD, L1-3. Increased functional connectivity within several networks associated with alcohol intake. Differences in total grey matter volume were observed in those drinking as little as 7–14 units (56–112 g) compared to <7 units weekly. Relationships were robust against unobserved confounding. Frequent binging, higher blood pressure and BMI were associated with steeper associations between alcohol and total grey matter volume. In contrast, alcohol beverage type consumed appeared to have little significance.

Non-drinkers comprised a higher proportion of females, higher mean material deprivation and lower educational qualifications compared with drinkers. Similar patterns have been observed in the wider UK population (Statistics OFN, 2017). The latest Office of National Statistics (ONS) data found higher proportions of females, those with lower income and no formal educational qualifications amongst the teetotaler group. The one discrepancy is that in the ONS data a lower proportion of teetotalers had never smoked, whereas in this UKB sample lower smoking was reported amongst non-drinkers.

Negative associations between alcohol and grey matter volume, as assessed with VBM, were spatially extensive. Older studies of non-dependent alcohol consumption and brain MRI have been somewhat conflicting (Topiwala and Ebmeier, 2018). Whilst some studies have reported lower white matter lesions and infarcts with light to moderate intake (den Heijer et al., 2004), others have reported higher atrophy (Mukamal et al., 2001) or lower grey matter volume in frontal, temporal and parietal lobes (Ewing et al., 2014). Our findings corroborate widespread associations with alcohol previously reported in the UKB in frontal, parietal and insular cortices, temporal and cingulate regions, putamen, amygdala and the brain stem (Evangelou et al., 2021, Daviet et al., 2022). In this UKB sample the strongest subcortical associations with alcohol we and others (Evangelou et al., 2021, Daviet et al., 2022) observed were negative with thalamus volume. Whilst we previously observed inverse associations between alcohol localized to hippocampal size in a separate smaller cohort sample (Topiwala et al., 2017), associations with hippocampal volume in UKB appear to be weaker. There is a great disparity in sample sizes, and therefore statistical power to detect small effects, between our previous analyses in Whitehall II (Topiwala et al., 2017) and studies in UKB. Daviet et al. found associations with alcohol in >90% of grey matter regions examined (Daviet et al., 2022), whereas Evangelou et al. reported a much narrower spatial distribution than we found, limited to cingulate, orbitofrontal regions, insula and thalami (Evangelou et al., 2021). Methodological differences may also play a role in explaining differing results. For example, our analyses have used FSL software in contrast to Evangelou et al. (Evangelou et al., 2021) who used SPM. Modulation (full vs. non-linear only), smoothing kernel (4.6–8 mm) and multiple comparison correction methods also differ. Daviet et al. (Daviet et al., 2022) examined IDPs derived from parcellations from atlases rather than voxel-based analyses used in this study and previously (Evangelou et al., 2021). There are potential ramifications of choices we made in our analysis pipelines. For example, we employed a small amount of spatial smoothing which is more sensitive to smaller anatomical differences and potentially less sensitive to differences of larger spatial extent. As cited previously, we found no evidence against associations with alcohol being monotonic (Evangelou et al., 2021). Furthermore, the observed widespread grey matter volume-alcohol associations in the current study persisted after additional sensitivity analyses not performed in previous studies, including adjustment for additional image-related confounders, and dividing abstainers into previous and never drinkers. This gives more confidence to the interpretation that alcohol is responsible for the brain structure associations, rather than other confounds known to impact brain measures (Alfaro-Almagro et al., 2021). Findings were unchanged when excluding previous drinkers. This lessens the possibility that individuals who have stopped drinking due to ill health, so called ‘sick quitters’, could be elevating the risk of non-drinkers (Marmot and Brunner, 1991), therefore underestimating the risks of drinking. A small number of voxels had positive associations with alcohol intake. The post-hoc analyses of lingual volume IDPs did not have convergent results, in fact negative associations with alcohol were observed as has been found by others (Daviet et al., 2022). The positive associations observed in the VBM analyses may have resulted from misregistrations, not practical to manually check at this scale. This is indirectly supported by the opposite findings using the FreeSurfer IDPs which are thought to be less prone to such problems. Additionally, many of the voxels with positive associations with alcohol in the VBM analyses were bordering CSF spaces and sinuses or supratentorial meninges. This raises the possibility of partial volume effects, although on visualization they appeared cortical. Smaller total grey matter volume was observed in participants drinking>7 units of alcohol weekly. This level of drinking is lower than currently defined as ‘low risk’ by the UK drinking guidelines (<14 units weekly) (Care DOHaS, 2016). Within UKB, others have observed associations with structural MRI at 7–14 units weekly and above (Daviet et al., 2022). Outside UKB, to our knowledge the lowest alcohol intake associated with MRI markers has been 14 units weekly (Topiwala et al., 2017). We hypothesise that the greater sample size of UKB enables detection of smaller effects. Whilst the effect size of the alcohol-total grey matter association was small in comparison to age, it was largest of the modifiable risk factors examined in this sample, making it relevant to public health.

Lower fractional anisotropy and mode, and higher mean and radial diffusivity were associated with higher alcohol consumption in this study. These findings are suggestive of alterations in white matter microstructure such as loss of myelin or axonal membranes (Friedrich et al., 20202020, Winklewski et al., 2018). The widespread associations we observed corroborate findings by another UKB study that examined DTI IDPs and also found associations with alcohol across many IDPs (Daviet et al., 2022). Furthermore, both studies highlighted strongest effects in the fornix. Previous studies reported more localized associations with alcohol in the corpus callosum (Topiwala et al., 2017) and corticospinal tracts (Evangelou et al., 2021). Somewhat surprisingly, a third UKB study did not find widespread associations between alcohol and diffusion IDPs (Evangelou et al., 2021). In fact, they observed positive associations between alcohol and corticospinal tract FA, which they suggest relate to crossing fibres. Again, methodological differences may explain these discrepancies. Evangelou used a smaller UKB sample (∼9000 participants vs. > 25,000 here and in Daviet et al.) thus had lower statistical power to detect small effects. Whilst our previous study (Topiwala et al., 2017) used, as in the current study, tract-based spatial statistics which allows examination of white matter microstructure in a finer grained manner, Daviet et al. (Daviet et al., 2022) and Evangelou et al. (Evangelou et al., 2021) examined multiple fibre tract IDPs. Correcting for global mean FA, associations between alcohol and FA appear less widespread. Adjustment for cardiovascular risk factors did not make a material difference to the strength of the associations, suggesting they were neither confounders nor on the causal pathway in our sample. Exploration of these factors was important, given that alcohol has known effects on cardiovascular risk (Roerecke et al., 2017, De Oliveira et al., 2000) and cardiometabolic disease has been highlighted as a mediator of alcohol-dementia relationships previously (Sabia et al., 2018).

This is the largest study, to our knowledge, of non-dependent alcohol consumption and functional connectivity. Higher alcohol intake was associated with increased functional connectivity within the default mode, attention, central executive and salience networks, and lower connectivity within the visual network. Of the few studies in alcohol dependent individuals, reduced functional connectivity in visual, executive, salience and default mode networks has been reported (Chanraud et al., 2011). Many factors could underlie the observed associations between alcohol intake and functional connectivity. Associations between connectivity and baseline alcohol intake could be the result of a participants’ chronic exposure to alcohol and subsequent neural changes (Fairbairn et al., 2021). Alternatively, higher weekly consumption could theoretically associate with intermittent mild symptoms or subclinical (minimally recognizable clinical findings) alcohol withdrawal (Jung and Metzger, 2010). These symptoms could include nausea, tremor, anxiety, higher blood pressure and pulse, amongst others.

The negative associations of binging with total grey matter volume is in keeping with other health outcomes, including mortality (Kauhanen et al., 1997), breast cancer (White et al., 2017), and cardiovascular disease (Mckee and Britton, 1998). Peak ethanol levels are higher during a binge. Binging followed by abstinence can precipitate alcohol withdrawal (Day and Daly, 2022). Withdrawal increases glutamate release (Hermann et al., 2012), microglial activation, inflammatory cytokines (Marshall et al., 2013) and can lead to brain injury (Jung and Metzger, 2010). Repeated binging-withdrawal cycles could magnify effects (Brown et al., 1988). Whilst most of our subjects were not alcohol-dependent, many reported binges, potentially resulting in subclinical withdrawal. Given the marked consequences for the brain of withdrawal, this represents a potential explanation for our observed interaction between alcohol intake and binging frequency in predicting grey matter volume.

Some studies have postulated protective effects of moderate drinking are strongest in wine (Gronbaek et al., 1995), due to polyphenols levels in grape skins. Our findings support the hypothesis that it is ethanol itself that is on the causal pathway of brain effects. Associations of wine-drinking with higher educational level and socioeconomic status may explain apparent health benefits (Mortensen et al., 2001).

We observed steeper associations between alcohol consumption and total grey matter volume in those with higher blood pressure and BMI. Higher blood pressure and BMI have been previously implicated in reducing grey matter volumes (Qizilbash et al., 2015, Beauchet et al., 2013) but their interaction with alcohol has not been explored. One mechanistic hypothesis is that hypertension may facilitate diffusion of ethanol throughout brain tissue. Animal models have demonstrated dysfunction of the blood brain barrier (BBB) in hypertension (Mackenzie et al., 1976, Biancardi et al., 2014). ApoE4 can also break down the BBB (Montagne et al., 2020), and therefore could hypothetically facilitate ethanol diffusion. We found no significant interaction between alcohol consumption and ApoE4, perhaps because of limited power due to the small number of E4 homozygotes. Animal models have shown synergistic effects of obesity and alcohol on steatohepatitis (Xu et al., 2011). BMI may increase the adverse impact of alcohol via the generation of toxic ceramides through a liver-brain axis (De La Monte et al., 2009). Ceramides have been linked to hippocampal atrophy (Kim et al., 2017) and risk of Alzheimer’s disease (Mielke et al., 2012). Alternatively, obesity could impair the intestinal barrier, facilitating bacterial endotoxin entry and pro-inflammatory cascades (Yan and Schnabl, 2012, Frazier et al., 2011, Brown, 2019).

MRI measures were associated with cognitive test performance, but direct associations between alcohol and cognition were only observed amongst participants with lower education. This could reflect a protective effect of education in line with cognitive reserve theory, or ceiling effects of certain measures (tower rearranging) (Stern et al., 1999). MRI may be more sensitive to the effects of alcohol than behavioral measures, especially given UKB’s limited cognitive battery which does not cover certain domains known to be affected by alcohol, such as verbal fluency (Topiwala et al., 2017, Fawns-Ritchie et al., 2020, Nowakowska-Domagała et al., 2017). Additionally, an unmeasured confounder, positively correlated to ‘moderate’ drinking and cognition, such as premorbid IQ could mask associations (Topiwala et al., 2017).

### 4.1 Limitations

UKB was selective, hence the proportion of participants who reported drinking heavily, or had a diagnosis of alcohol dependence, was low. We cannot exclude possible residual confounding, but our sensitivity analyses demonstrated the low likelihood of this obviating the observed associations. The age when alcohol was self-reported limits the interpretation estimates to the impact of mid- to late-life consumption. Self-reported alcohol may suffer misclassification bias, including desirability bias with individuals underreporting their intake. However, self-report is the only realistically available method at scale (Conigrave et al., 2003) and is used in clinical practice. Random measurement error would bias associations towards the null, whereas desirability bias could overestimate associations at low levels of alcohol. Neuroimaging was cross-sectional and therefore we cannot examine the impact of alcohol on *changes* in brain measures over time. We are mindful of greater power to detect associations amongst wine drinkers than amongst spirit drinkers. As with any observational study, we cannot make causal claims about the directionality of associations between alcohol and neuroimaging markers. However, reverse causation is unlikely because the earliest detectable brain changes occur in the late 40′s, by which time there have usually been decades of alcohol exposure.

In this large neuroimaging sample, alcohol consumption was negatively associated with total grey matter volume, multiple markers of white matter microstructure and higher functional connectivity. Lower total grey matter volume amongst drinkers was evidenced even within current UK ‘low risk’ drinking guidelines (<14 units weekly). Because moderate drinking is highly prevalent, even small associations could have substantial population impact. A realistic estimate of the potential effects of population interventions has to wait for the results of randomized intervention studies. It remains unclear how duration of drinking affects associations, and whether particular life periods represent heightened vulnerability (Mewton et al., 2020). Studies in alcohol-dependent drinkers suggest at least some damage is reversible upon abstinence. We do not know whether the same follows for moderate intakes.

## Funding

AT is supported by a Wellcome Trust fellowship (216462/Z/19/Z) and KPE by the UK Medical Research Council (G1001354 & MR/K013351/1) and the European Commission (Horizon 2020 732592). This work was also supported by the Li Ka Shing Centre for Health Information and Discovery, NIH grant (TMS, TN: R01EB026859), and a Wellcome Trust award (TN: 100309/Z/12/Z).

## CRediT authorship contribution statement

**Anya Topiwala:** Conceptualization, Formal analysis, Funding acquisition, Investigation, Methodology, Visualization, Writing – original draft, Writing – review & editing. **Klaus P. Ebmeier:** Writing – original draft, Writing – review & editing. **Thomas Maullin-Sapey:** Formal analysis, Methodology, Writing – review & editing. **Thomas E. Nichols:** Formal analysis, Methodology, Visualization, Supervision, Writing – original draft, Writing – review & editing.

## Declaration of Competing Interest

The authors declare that they have no known competing financial interests or personal relationships that could have appeared to influence the work reported in this paper.
