# Supplementary material for: Alcohol consumption and MRI markers of brain structure and function: Cohort study of 25,378 UK Biobank participants
Source: Neuroimage Clin. 2022 May 28;35:103066. doi: 10.1016/j.nicl.2022.103066 (PMC9163992; doi:10.1016/j.nicl.2022.103066)
Supplement: Supplementary data 1 [file mmc1.docx]

**Supplementary methods**

Tabular variables were extracted from UKB files using FSL’s funpack [1].

**Alcohol consumption**

Glasses were converted to UK units as follows [2]: red or white wine = 1.7 units; fortified wine=1.2 units; pint = 2.4 units; spirits = 1 unit; other (e.g. alcopops) =1.2 units. Amounts were also converted to grams pure ethanol (1 UK unit=8g) to aid international comprehension and comparison. For monthly intake, units were divided by 4.3 to estimate a weekly amount. Amounts were summed across beverage types and weekly and monthly intakes to generate a total weekly alcohol unit intake used for further analyses. This weekly total was additionally divided into quintiles for selected analyses.

**MRI acquisition**

As part of the protocol [3] T1-weighted images were obtained using an MPRAGE sequence: TR=2000ms, TE=2.0ms, 208 sagittal slices, flip angle=8°, FOV=256mm, matrix=256x256, slice thickness=1.0mm (voxel size 1x1x1mm). Diffusion images were obtained using a spin-echo echo-planar sequence with 10 T2-weighted baseline volumes, 50b = 1000 s mm^-2^ and 50 b=2000 s mm^-2^ diffusion weighted volumes, with 100 diffusion-encoding directions and 2mm isotropic voxels. Resting state functional MRI was acquired with the following parameters: TE=39ms, TR=735ms, MB=8, R=1, flip angle=52°, 490 time points, 2.4x2.4x2.4mm voxel size.

**Further details about statistical analyses**

*Big Linear Toolbox* [4] - A missingness threshold of 80% was employed (i.e. voxels with recorded data for less than 80% of subjects were discarded from the analysis), and two T contrasts (positive and negative correlation with alcohol) and an F contrast were computed.

*Restricted cubic splines* – The effect of alcohol was parameterized with a cubic spline with 5 knots. Knots were placed at 5^th^, 25^th^, 50^th^, 75^th^ and 95^th^ percentiles (0.2, 1.9, 10.1, 20.3, 49.2 units) with the tails restricted to linearity (for stability). The knot number and position used are recommended in the literature and were empirically tested for this data using AIC criteria [5]. A spline model avoids the loss of power and arbitrary cut-points of categorization, and fits threshold effects better than polynomials. It offers a flexible approach to estimate the shape of the exposure-outcome curve which was of key interest in this study.

*Comparing alcohol effects on grey matter to alcohol effects –* Higher order age terms and age x sex interactions were orthogonalized with respect to the main linear age term. All were then entered into the regression model with grey matter as the dependent variable, together with other covariates. The effect size for 1 unit higher alcohol consumption over the study could then be compared with that for being 1 year greater in age at study baseline.

*Sensitivity analysis for unobserved confounding [6]* - The impact of an unobserved confounder depends on two measures: its association with brain health (the outcome) and its association with alcohol (the exposure of interest). The strength of these associations can be measured in partial R2. The robustness value is the level of outcome and exposure confounding (assumed to be equal for these purposes) required to zero-out the alcohol-brain health association if we actually could control for the unobserved confound. Plausible outcome and exposure confounding partial R2’s can be computed for known confounds. This allows the impact of an unobserved confound to be calibrated by the severity of the strongest existing confounds. We used age, sex and smoking (the strongest known confounders for grey matter volume and alcohol intake) for this purpose.

*Software details –* Restricted cubic splines were applied using the R package rms v.6.2-0*.* Spline models were plotted graphically using R’s predict function to show how each brain IDP (z-score) would be expected to change with alcohol consumption, keeping other independent variables at a fixed level. Sensitivity analysis for unobserved confounding used R’s sensemakr package. Standardised regression coefficients (generated by converting outcomes to z scores) for were plotted using R’s jtools package (v2.1.3). Manhattan plots (implemented in R’s qqman package v0.1.8) were used to display associations between ‘edges’ (functional connectivity between nodes) and alcohol intake.

1. Mccarthy P. funpack. 2021.

2. Clarke T-K, Adams MJ, Davies G, Howard DM, Hall LS, Padmanabhan S, et al. Genome-wide association study of alcohol consumption and genetic overlap with other health-related traits in UK Biobank (N= 112 117). Molecular psychiatry. 2017;22(10):1376-84.

3. Smith Stephen M A-aF, Miller Karla L. UK Biobank Brain Imaging Documentation. 2020(Version 1.8).

4. Maullin-Sapey TNTE. BLM toolbox for neuroimaging cluster and local usage

5. Jr HF. Regression Modeling Strategies. 2006.

6. Cinelli C, Hazlett C. Making sense of sensitivity: Extending omitted variable bias. Journal of the Royal Statistical Society: Series B (Statistical Methodology). 2020;82(1):39-67.
