# Supplementary material for: Alcohol consumption and MRI markers of brain structure and function: Cohort study of 25,378 UK Biobank participants
Source: Neuroimage Clin. 2022 May 28;35:103066. doi: 10.1016/j.nicl.2022.103066 (PMC9163992; doi:10.1016/j.nicl.2022.103066)
Supplement: Supplementary data 2 [file mmc2.docx]

**Index of supplementary figures and tables**

**Overview of analyses**

**Table 1:** Summary of analyses

**Descriptive analyses**

**Figure 1:** Flow chart of included participants

**Table 2:** Sociodemographic comparison of included versus imaged sample

**Figure 2:** Cumulative distribution of alcohol consumption

**Table 3:** Predictors of binging frequency

**Figure 3:** Cumulative distribution of alcohol consumption by beverage type

**Table 4:** Baseline characteristics by alcoholic beverage type

**T1 structural analyses**

**Figure 4:** VBM with additional imaging-related confounder adjustment

**Figure 5:** VBM, current drinkers

**Table 5:** Regression models with and without cardiovascular risk factors

**Figure 6:** Voxelwise variance of grey matter density explained by alcohol intake, all subjects

**Table 6:** Variance of grey matter volume explained by modifiable risk factors

**Table 7:** Effect sizes used to equate alcohol - grey matter volume associations with age – grey matter volume associations

**Figure 7:** Predicted grey matter IDP volumes according to alcohol intake

**Table 8:** Regression models for grey matter image-derived phenotypes (IDPs)

**Figure 8:** Sensitivity contour plot for alcohol-grey matter estimate

**Table 9:** Regression models predicting cross-sectional cognitive performance with grey matter volume

**Table 10:** Regression models predicting cross-sectional cognitive performance with alcohol

**Diffusion tensor imaging (DTI) analyses**

**Figure 10:** Tract-based spatial statistics (TBSS) – FA, image-related confounder adjustment

**Figure 11:** TBSS – FA, adjustment for global mean fractional anisotropy

**Figure 12:** TBSS, current drinkers

**Figure 13:** TBSS - mean diffusivity, all subjects

**Figure 14:** TBSS - mode, all subjects

**Figure 15:** TBSS – L1, all subjects

**Figure 16:** TBSS - L2, all subjects

**Figure 17:** TBSS - L3, all subjects

**Functional connectivity analyses**

**Table 11:** Key resting state networks and their constituent regions

**Table 12:** Networks within which functional connectivity associated with alcohol

**Figure 18:** Predicted resting state node amplitude according to alcohol intake

**Table 13:** Associations between alcohol and between network connectivity

**Figure 19:** Associations between alcohol and between network connectivity (effect sizes)

**Figure 20:** Associations between within network connectivity and cognitive test performance

**Figure 21:** Associations in Figure 20 adjusted for alcohol

**Pre-specified subgroup analyses**

**Table 14:** Regression models including significant interactions

**Table 15:** Regression models of blood pressure-alcohol interactions controlling for antihypertensives

**Table 16:** Regression models including non-significant interactions

**Table 17:** Regression models examining binge-drinking frequency

**Figure 22:** Comparison of wine, beer and spirit drinking in predicting grey matter

**Figure 23:** Blood pressure and body mass index modify alcohol-grey matter associations

|  | **Analysis** | **Independent variable of interest** | **Outcome** | **Confounds** |
| --- | --- | --- | --- | --- |
| **Main** | Voxel-based morphometry | Weekly alcohol | Grey matter volume | Standard |
|  | Tract-based spatial statistics | Weekly alcohol | Fractional anisotropy, mean diffusivity, mode, L1-3 | Standard |
|  | Regression | Weekly alcohol | Grey matter volume image-derived phenotypes (total, hippocampus, thalamus, amygdala putamen) | Standard + estimated intracranial volume |
|  | Regression | Weekly alcohol | Resting state functional MRI *within* network connectivity | Standard |
|  | Regression | Weekly alcohol | Resting state functional MRI *between* network connectivity | Standard |
|  | Regression | Total grey matter volume | Cognitive performance | Standard |
|  | Regression | Resting state functional MRI *within* network connectivity | Cognitive performance | Standard |
| **Sensitivity** | Voxel-based morphometry / tract-based spatial statistics /image-derived phenotype | Weekly alcohol (excluding non-drinkers) | Grey matter volume/ fractional anisotropy /grey matter volumes | Standard + estimated intracranial volume (volume analyses) |
|  | Image-derived phenotype | Weekly alcohol | Total grey matter volume | Standard excluding blood pressure, cholesterol + estimated intracranial volume |
|  | Voxel-based morphometry, tract-based spatial statistics | Weekly alcohol | Grey matter volume/ fractional anisotropy | Standard+ image-related + estimated intracranial volume |
|  | Tract-based spatial statistics | Weekly alcohol | Fractional anisotropy | Standard+ global mean fractional anisotropy |
|  | Regression | Weekly alcohol | Total grey matter volume | Standard + estimated intracranial volume |
| **Subgroup** | Regression | Alcohol x age/sex/blood pressure/body mass index/ApoE4 | Total grey matter volume | Standard + estimated intracranial volume |
|  | Regression | Weekly alcohol (wine vs. beer vs. spirits) | Total grey matter volume | Standard + estimated intracranial volume |
|  | Regression | Frequency of binge drinking | Total grey matter volume | Standard+ total alcohol volume consumed + estimated intracranial volume |

**Table 1: Summary of analyses grouped by type. Cognitive performance was assessed at the time of scanning on: pairs matching, digit span, matrix puzzle, Trail-making test, tower correlation, reaction time, prospective memory and fluid intelligence. ‘Standard’ confounds were: age, sex, smoking, educational qualifications, systolic and diastolic blood pressure, body mass index, Townsend Deprivation Index, Metabolic Equivalent Task minutes weekly, diabetes mellitus, depression, total and high-density cholesterol, imaging site. Additional image-related confounds included: head motion, table position, scanner acquisition parameters (site, scanner software, protocol, scan ramp, head coil).**

**Descriptive analyses**

UK Biobank participants who attended imaging visit

N=48,702

Structural abnormality, missing alcohol or confounder data. N=23,324

Subjects in VBM analyses, with usable images, complete alcohol and confounder data

N=25,378

N=550

TBSS could not be run

N=1348

Subjects in TBSS analyses

N=24,030

Missing FIRST volumes or diffusion parameters

N=1776 write.csv(idp2, "/well/nichols/users/wld330/imaging_48k/brain_idp/idp_quant.csv")

Subjects included in structural IDP analyses

N=22,253

Missing resting state fMRI data

N=4667

Subjects included in functional IDP analyses

N=17,587

Missing data on binge-drinking

N=4775

Subjects included in binge-drinking analysis

N=12,817

**Figure 1: Flow chart of participants included in analysis. Abbreviations: VBM – voxel-based morphometry, TBSS –tract-based spatial statistics, IDP – image-derived phenotype, fMRI – functional magnetic resonance imaging.**

| **Sociodemographic factor** | **Sample attending imaging visit (n=48,702)** | **VBM analysis group (n=25,378)** | **Group differences** |
| --- | --- | --- | --- |
| Age at baseline, *years* | 55.19 (7.57) | 55.10 (7.53) | 9.09 (-0.02 to 0.20), t=1.54, p=0.12 |
| Sex, *females* | 25,150 (51.64) | 12,254 (48.29) | 3.35 (2.59 to 4.11), p<0.0001 |
| University or college degree | 22,189 (45.56) | 12,377 (48.77) | 3.21 (2.45 to 3.97), p<0.0001 |
| No educational qualifications | 3214 (6.60) | 1384 (5.45) | 1.15 (0.79 to 1.50), p<0.0001 |

**Table 2: Sociodemographic comparison of sample attending the imaging visit with the sample with sufficient data to be included in the voxel-based analysis (VBM). Mean (standard deviation) is given for age, and number (proportion) for sex and educational qualifications. Group differences (95% confidence intervals) were calculated using a t-test for age, and test of proportions for categorical variables.**

**
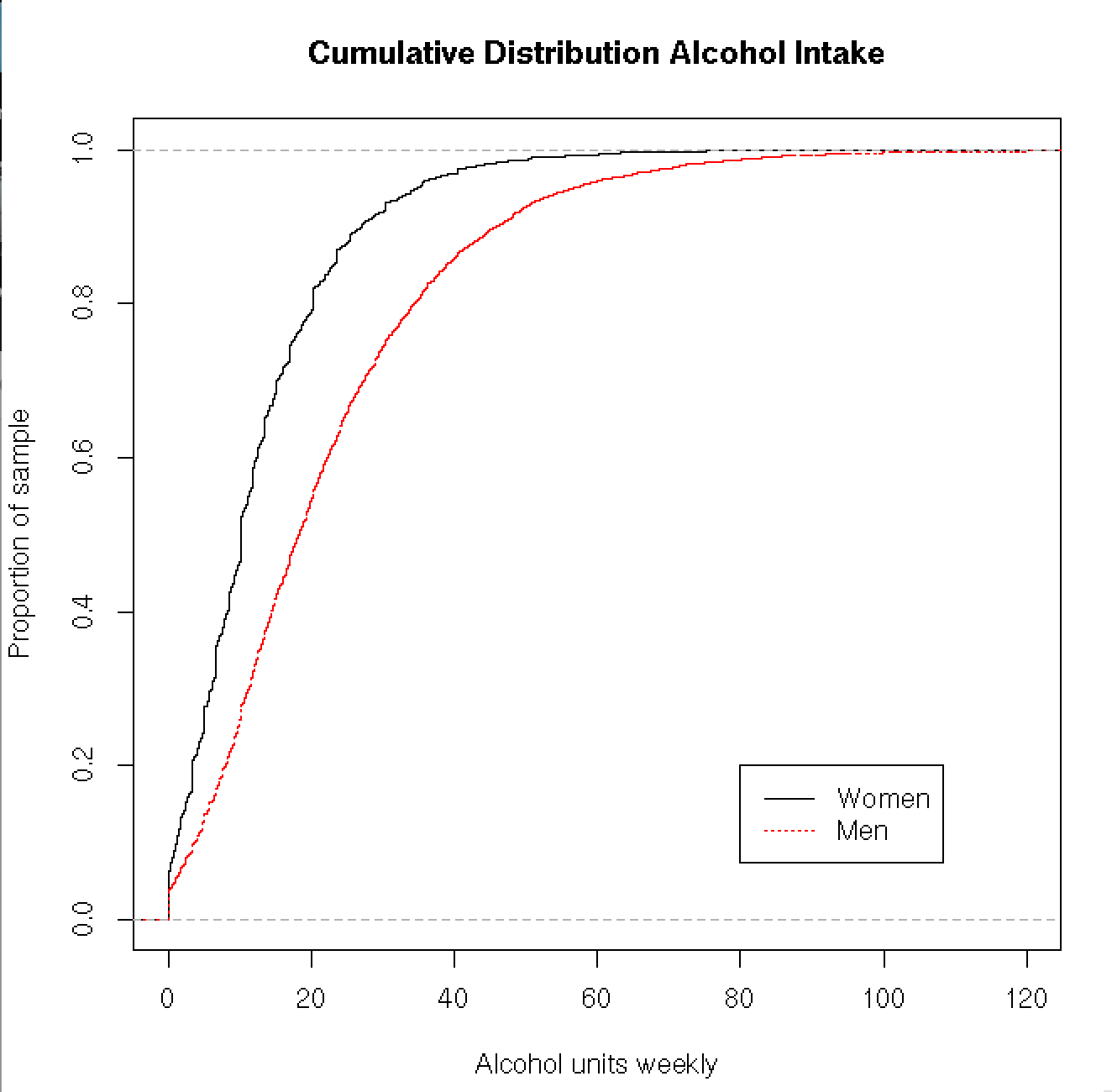
**

**Figure 2: Cumulative distribution of weekly alcohol consumption separately by sex, weekly units. N=25,378.**

| **Sociodemographic factor** | **Coefficient** | **95% confidence interval** | **P value** |
| --- | --- | --- | --- |
| Age, *years* | -0.07 | -0.07 to –0.06 | 4.40 x 10^-210^ |
| Sex | 0.21 | 0.14 to 0.27 | 4.60 x 10^-10^ |
| Current vs. never smoker | 0.52 | 0.39 to 0.65 | 3.30 x 10^-15^ |
| University or college degree vs. no educational qualifications | 0.51 | 0.35 to 0.67 | 1.00 x 10^-9^ |
| Townsend Deprivation Index | 0.02 | 0.01 to 0.03 | 3.58 x 10^-4^ |

**Table 3: Predictors of alcohol binging frequency. Binging is defined as >6 units (48g) alcohol in one drinking episode. Models were adjusted for weekly alcohol intake in units. N=14,685. Estimates generated using ordinal logistic regression, with binging frequency as the dependent variable, and each sociodemographic factor entered separately. P values were generated by comparing the t-value against the standard normal distribution.**

**
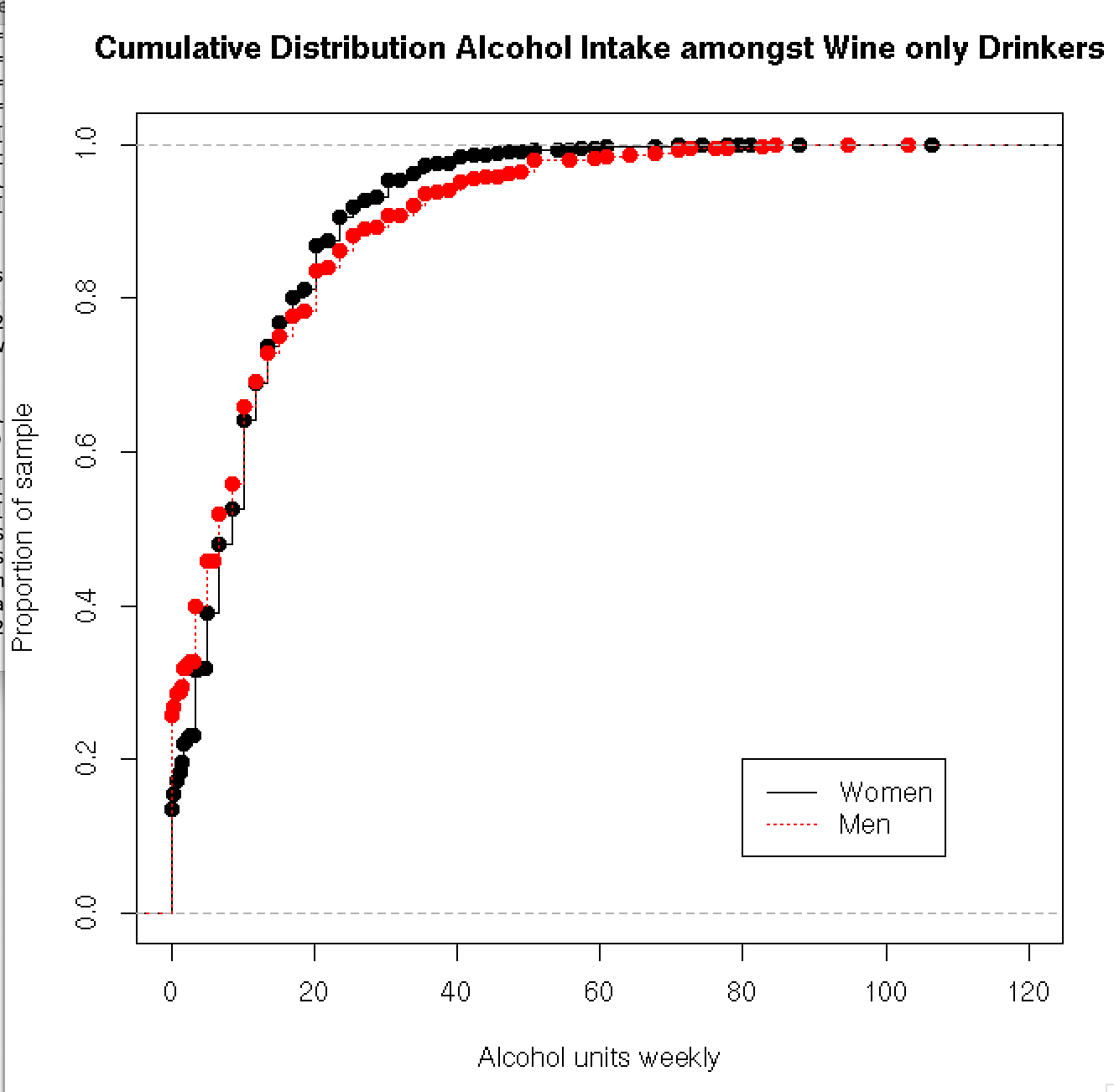

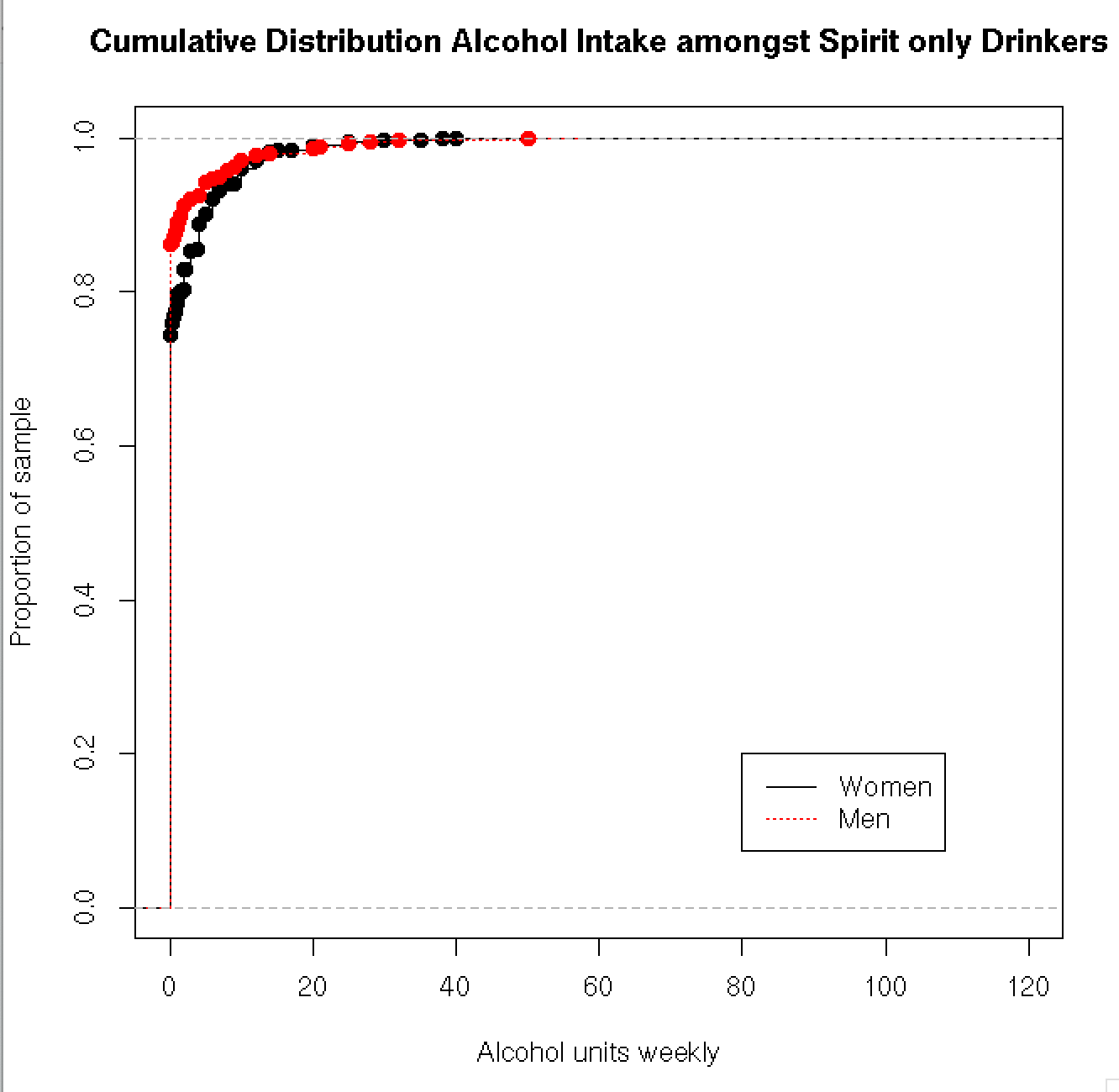
**

**
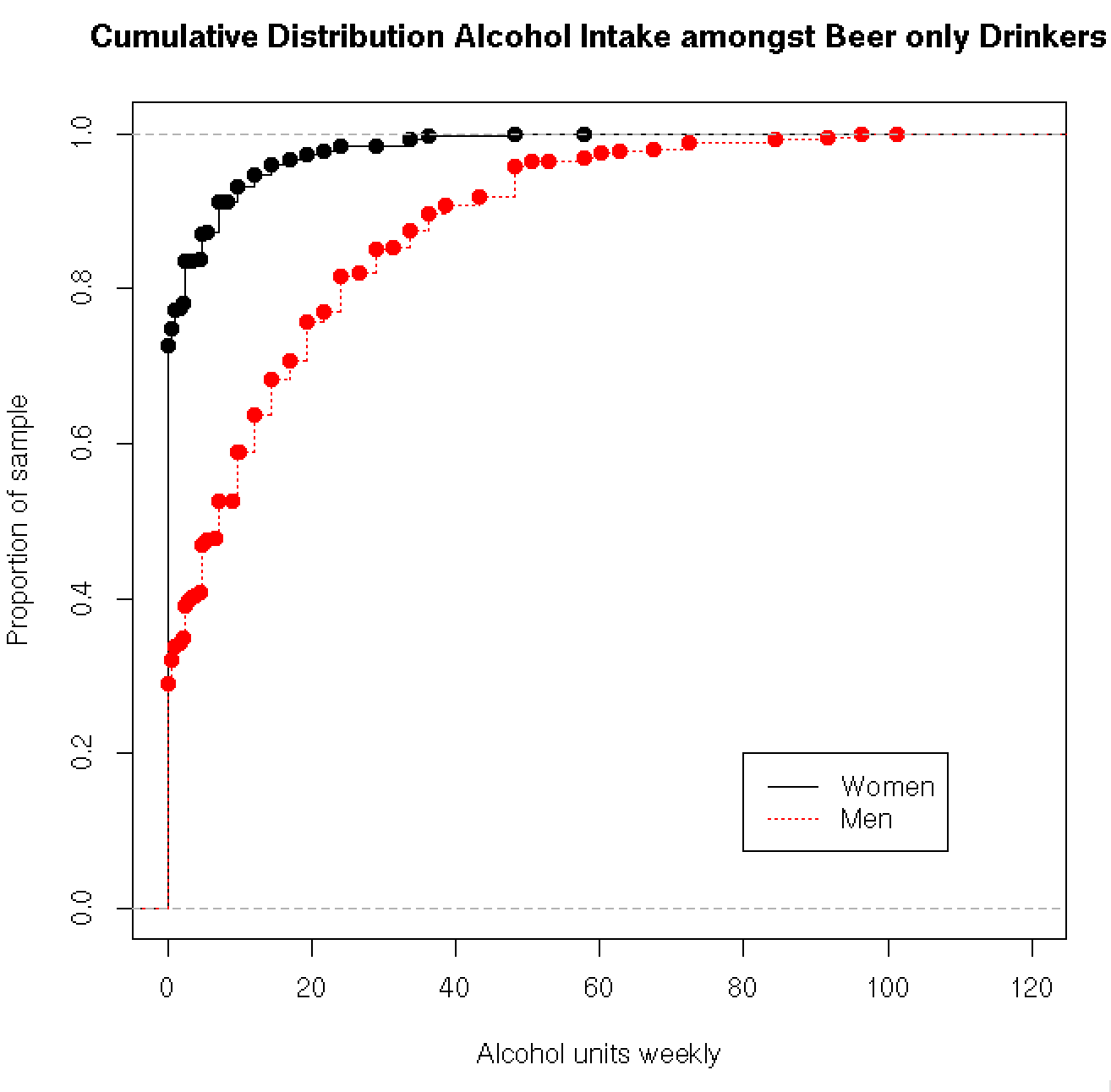
**

**Figure 3: Cumulative distribution of alcohol intake in weekly units for subjects drinking solely wine (n=5080), beer (n=1193) and spirits (n=329).**

|  | **Wine drinkers (N=5080)** | **Beer drinkers (N=1193)** | | **Spirits drinkers (N=329)** | | **Group differences** | |
| --- | --- | --- | --- | --- | --- | --- | --- |
| **Age, *years*** | 55.24 (7.29) | | 53.39 (7.64) | | 54.92 (8.00) | | F(1,6600)=28.28,p=1.09x10^-7^ |
| **Sex, N(%) *female*** | 3996 (78.66) | | 280 (23.47) | | 259 (78.72) | | X^2^=1384.6, df=2,p<2.2x10^-16^ |
| **Educational qualifications, *none*** | 232 (4.57) | | 122 (10.23) | | 43 (12.07) | | X^2^=375.55, df=12, p<2.2x10^-16^ |
| ***A levels*** | 689 (13.56) | | 150 (12.57) | | 42 (12.77) | |  |
| ***University or college degree*** | 2262 (52.40) | | 381 (31.94) | | 103 (31.32) | |  |
| **BMI, *kg/m^2^*** | 25.69 (4.05) | | 27.26 (4.27) | | 26.69 (4.46) | | F(1,6600)=107.9,p<2x10^-16^ |
| **SBP, *mmHg*** | 134.88 (19.01) | | 138.35 (17.67) | | 133.97 (18.37) | | F(1,6600)=8.62,p=0.003 |
| **DBP, *mmHg*** | 80.11 (10.31) | | 82.86 (10.49) | | 79.78 (10.42) | | F(1,6600)=22.18, p=2.53x10^-6^ |
| **Total cholesterol, *mmol/L*** | 5.79 (1.06) | | 5.60 (1.08) | | 5.80 (1.10) | | F(1,6600)=10.71,p=0.001 |
| **Metabolic Equivalent Task, *minutes weekly*** | 95 (93) | | 110 (130) | | 100 (120) | | X^2^=24.09, df=2, p=5.88x10^-6^ |
| **Townsend Deprivation Index** | -2.14 (2.54) | | -1.44 (2.80) | | -1.64 (2.77) | | F(1,6600)=57.29, p=4.28x10^-14^ |
| **Smoking status, *never*** | 3257 (64.11) | | 665 (55.74) | | 202 (61.40) | | X^2^=83.62, df=4,p<2.2x10^-16^ |
| ***previous*** | 1608 (31.64) | | 406 (34.03) | | 97 (29.48) | |  |
| ***current*** | 215 (4.23) | | 122 (10.23) | | 30 (9.12) | |  |
| **Diabetes mellitus, *N(%)*** | 159 (3.13) | | 85 (7.12) | | 17 (5.17) | | X^2^=41.95, df=2,p<7.77x10^-10^ |

**Table 4: Baseline characteristics (mean(standard deviation), median(interquartile range) or percentage (proportion)) according to alcohol beverage consumed. Abbreviations: BMI – body mass index, SBP – systolic blood pressure, DBP – diastolic blood pressure. Selected qualification categories are displayed. Group differences were calculated using one-way ANOVA for continuous variables and chi-square test for categorical variables.**

**Grey matter analyses**

**
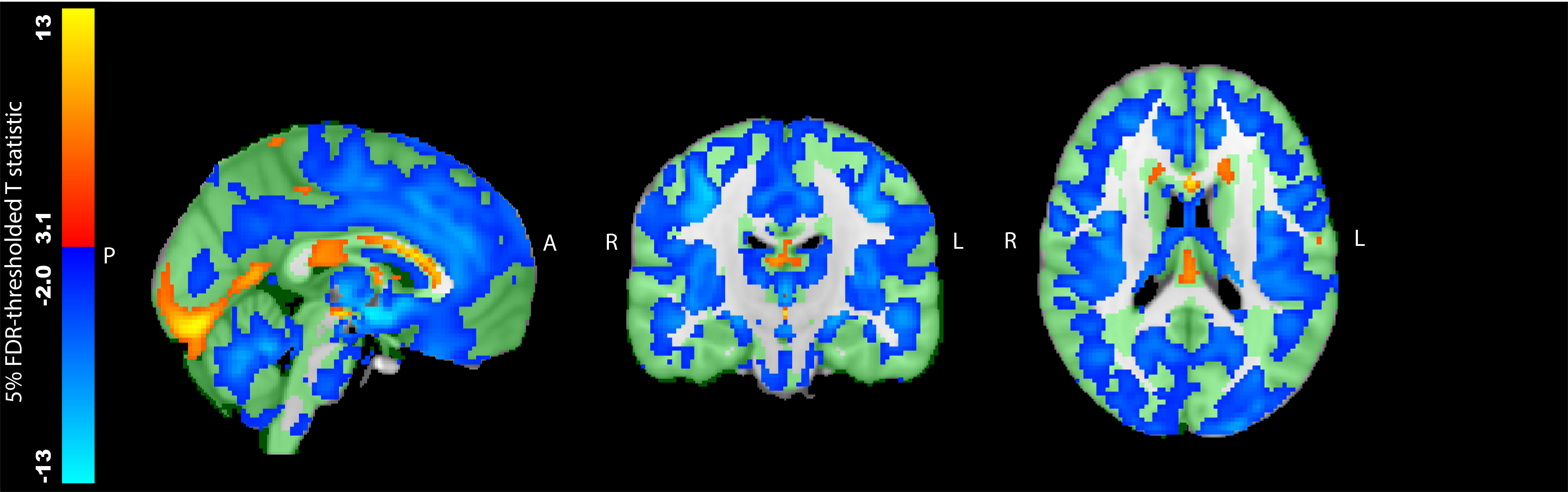
**

**Figure 4: Associations between weekly alcohol intake and grey matter density generated using voxel-based morphometry, with additional imaging-related confounder adjustment (n=22,536). T statistics are thresholded at 5% False Discovery Rate (0.025 threshold on uncorrected p values for negative association and 0.001 uncorrected p values for positive associations). Study specific grey matter mask shown in green. Adjusted for: age, sex, age^2^, age^3^, age x sex, age^2^ x sex, imaging site, blood pressure, total cholesterol, high-density lipoprotein, diabetes mellitus, smoking, body mass index, Metabolic Equivalent Task minutes weekly, Townsend Deprivation Index, depression, educational qualifications, estimated intracranial size, head motion, table position, acquisition parameters.**

**
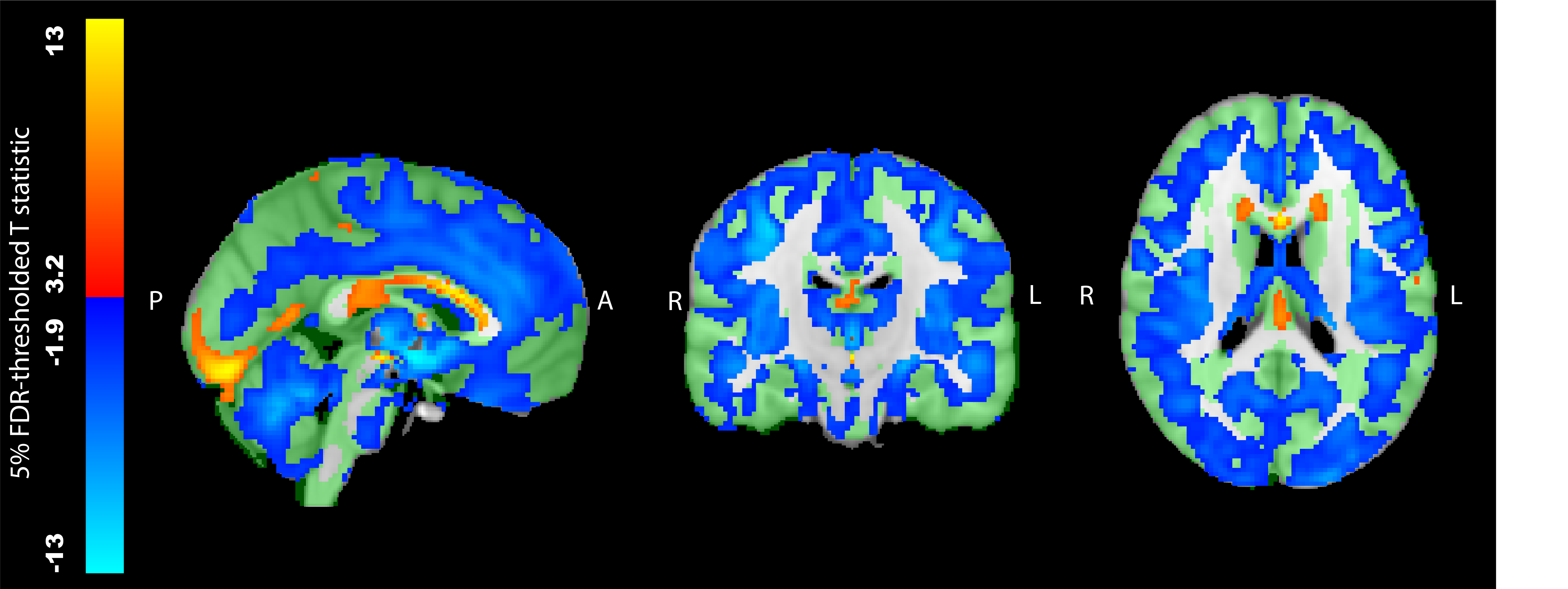
**

**Figure 5: Associations between weekly alcohol intake and grey matter volume generated using voxel-based morphometry, current drinkers only (n=24,069). T statistics are thresholded at 5% False Discovery Rate (0.028 threshold on uncorrected p values for negative association and 0.0007 uncorrected p values for positive associations). Study specific grey matter mask shown in green. Adjusted for: age, sex, age^2^, age^3^, age x sex, age^2^ x sex, imaging site, blood pressure, total cholesterol, high-density lipoprotein, diabetes mellitus, smoking, body mass index, Metabolic Equivalent Task minutes weekly, Townsend Deprivation Index, depression, educational qualifications, head motion, table position, acquisition parameters.**

|  | | |
| --- | --- | --- |
|  | **Reduced model** | **Full model** |
|  | | |
| **Alcohol, *units weekly*** | -0.10^**^ (-0.11, -0.09) | -0.10^**^ (-0.11, -0.09) |
| **SBP, *mmHg*** |  | 0.02^**^ (0.01, 0.04) |
| **DBP, *mmHg*** |  | -0.04^**^ (-0.06, -0.03) |
| **Non-HDL cholesterol, *mmol/L*** |  | 0.02^**^ (0.01, 0.03) |
| **Age, *years*** | 0.96 (-0.45, 2.36) | 1.07 (-0.33, 2.47) |
| **Sex** | -0.63^*^ (-1.12, -0.15) | -0.71^**^ (-1.20, -0.23) |
| **Age^2^** | -2.71^*^ (-5.54, 0.11) | -2.96^*^ (-5.78, -0.14) |
| **Age^3^** | 1.16 (-0.28, 2.59) | 1.28 (-0.16, 2.72) |
| **Age x sex** | 0.97 (-0.06, 1.99) | 1.12^*^ (0.09, 2.14) |
| **Age^2^ x sex** | -0.38 (-0.94, 0.18) | -0.45 (-1.01, 0.11) |
| **Higher degree vs. no educational qualification** | -0.02 (-0.06, 0.02) | -0.02 (-0.06, 0.03) |
| **A levels vs. no educational qualification** | 0.00 (-0.04, 0.05) | 0.01 (-0.04, 0.06) |
| **GCSEs vs. no educational qualification** | -0.01 (-0.06, 0.04) | -0.01 (-0.05, 0.04) |
| **CSEs vs. no educational qualification** | 0.03 (-0.03, 0.09) | 0.03 (-0.03, 0.09) |
| **NVQ vs. no educational qualification** | -0.01 (-0.07, 0.05) | -0.01 (-0.06, 0.05) |
| **Professional qualification vs. no educational qualification** | 0.05 (-0.01, 0.11) | 0.05 (-0.01, 0.11) |
| **Site 1** | -0.15^**^ (-0.18, -0.12) | -0.15^**^ (-0.18, -0.12) |
| **Site 2** | -0.12^**^ (-0.14, -0.10) | -0.12^**^ (-0.14, -0.10) |
| **Previous vs. never smoker** | -0.06^**^ (-0.08, -0.04) | -0.06^**^ (-0.08, -0.04) |
| **Current vs. never smoker** | -0.15^**^ (-0.19, -0.11) | -0.16^**^ (-0.19, -0.12) |
| **TDI** | -0.02^**^ (-0.03, -0.01) | -0.02^**^ (-0.03, -0.01) |
| **Metabolic Equivalent Task, *minutes weekly*** | 0.00 (-0.01, 0.01) | 0.00 (-0.01, 0.01) |
| **Diabetes mellitus** | -0.27^**^ (-0.32, -0.23) | -0.27^**^ (-0.31, -0.22) |
| **Estimated intracranial volume** | 0.30^**^ (0.28, 0.31) | 0.30^**^ (0.28, 0.31) |
| **BMI** | -0.06^**^ (-0.07, -0.05) | -0.05^**^ (-0.06, -0.04) |
| **Constant** | 0.10^**^ (0.06, 0.14) | 0.10^**^ (0.06, 0.14) |
| **N** | 22,254 | 22,254 |
| **R^2^** | 0.51 | 0.51 |
| **Adjusted R^2^** | 0.51 | 0.51 |
| **Residual Std. Error** | 0.70 (df = 22231) | 0.70 (df = 22228) |
| **F Statistic** | 1,041.75^**^ (df = 22; 22231) | 921.30^**^ (df = 25; 22228) |
|  | | |

**Table 5: Regression models predicting total grey matter volume, with and without cardiovascular risk factors affected by alcohol (standardised estimates). Abbreviations: TDI – Townsend Deprivation Index, SBP – systolic blood pressure, DBP – diastolic blood pressure, BMI – body mass index, HDL – high-density lipoproteins, df – degrees of freedom. ^*^uncorrected p < .05; ^**^uncorrected p < .01.**

**
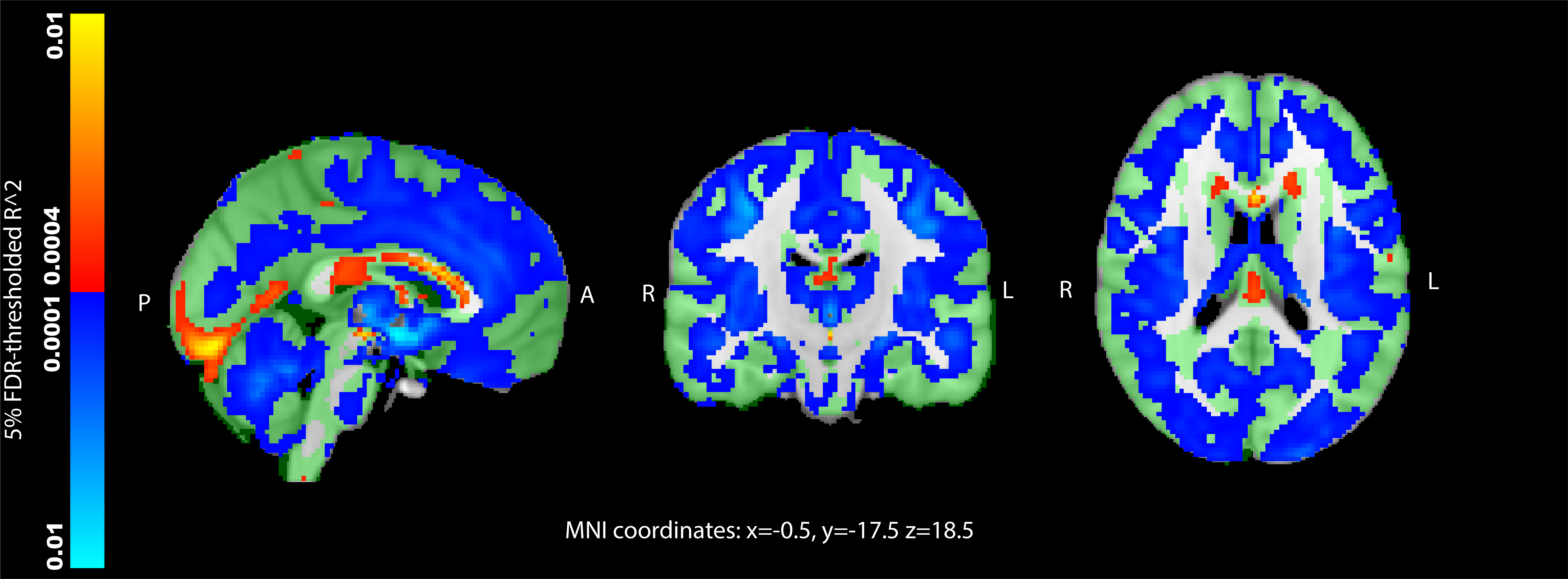
**

**Figure 6: Variance of grey matter volume explained by weekly alcohol intake (partial R^2^ values) shown voxel-wise N=25,378 subjects. Partial R^2^ are thresholded at 5% False Discovery Rate (0.028 threshold on uncorrected p values for negative association and 0.001 uncorrected p values for positive associations). Study specific grey matter mask shown in green. Adjusted for: age, sex, age^2^, age^3^, age x sex, age^2^ x sex, imaging site, blood pressure, cholesterol, high-density lipoprotein, diabetes mellitus, smoking, body mass index, Metabolic Equivalent Task minutes weekly, Townsend Deprivation Index, depression, educational qualifications, head motion, table position, acquisition parameters.**

| **Risk factor** | **Partial R^2^ explained by risk factor (%)** |
| --- | --- |
| **Alcohol consumption** | **0.02** |
| Smoking status | 0.004 |
| Body mass index | 0.005 |
| Systolic blood pressure | 0.0004 |
| Diastolic blood pressure | 0.002 |
| Non-high-density cholesterol | 0.0008 |

**Table 6: Partial R^2^ for modifiable risk factors. N=22,254 subjects. Partial R^2^ represents the squared correlation between the risk factor and total grey matter volume after adjusting both exposure and outcome for all other risk factors listed in column 1, and: age, sex, imaging site, estimated intracranial volume, diabetes mellitus, Townsend Deprivation Index, educational qualifications.**

|  | **Estimate (95% CI)** |
| --- | --- |
| **Alcohol** | -6.37x10^-3^ (-7.04 to -5.69x10^-3^) |
| **Age (main, linear)** | -7.73x10^-2^ (-7.87x10^-2^ to -7.58x10^-2^) |
| **Age^2^ (orthogonalized w.r.t age)** | -6.50e-04 (-8.27x10^-4^ to -4.73x10^-4^) |
| **Age^3^ (orthogonalized w.r.t age)** | 2.18e-05 (-6.80x10^-7^ to 4.42x10^-5^) |
| **Age x sex (orthogonalized w.r.t age)** | 0.11 (8.04x10^-3^ to 0.14) |
| **Age^2^ x sex (orthogonalized w.r.t age)** | 0.32 (-6.71x10^-4^ to 3.05x10^-5^) |

**Table 7: Effect sizes used to equate alcohol – total grey matter volume associations with age – grey matter volume associations. Estimates generated from a regression model adjusted for: age (linear), age^2^, age^3^, age*sex, age^2^*sex, imaging site, estimated intracranial volume, body mass index, blood pressure, Townsend Deprivation Index, cholesterol, high-density lipoprotein, diabetes mellitus, educational qualifications, smoking and Metabolic Equivalent Task minutes weekly. Abbreviations: w.r.t – with respect to, CI – confidence interval.**

**
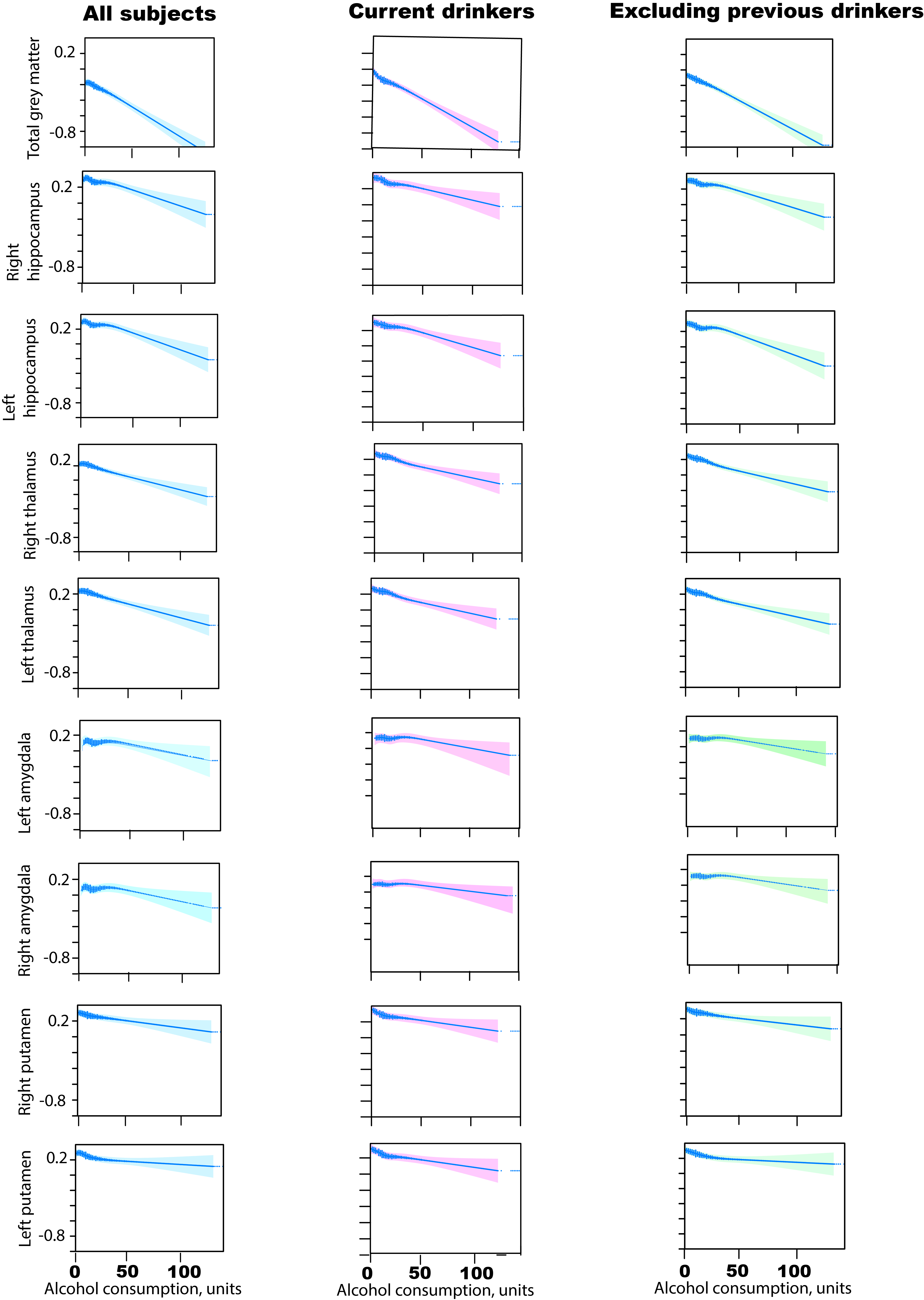
**

**Figure 7: Predicted change in selected brain volumes according to weekly alcohol intake in units for a) all subjects (n=22,253) b) current drinkers (excluding never and previous drinkers, n=18,398) (c) excluding previous drinkers (n=19,409). Predictions are based on regression models with alcohol (restricted cubic splines fitted with knots at 5^th^, 25^th^, 50^th^, 75^th^ and 95^th^ percentiles) as an independent variable and standardized brain volume as the dependent variable. Models are adjusted for: age, sex, age^2^, age^3^, age x sex, age^2^ x sex, blood pressure, Townsend Deprivation Index, educational qualifications, smoking, body mass index, non-HDL cholesterol, diabetes mellitus, estimated intracranial volume, Metabolic Equivalent Task minutes weekly. 95% confidence intervals are shaded. Grams conversion: 50u=400g, 100u=800g.**

|  | **Total grey matter** | **Hippocampus, R** | **Hippocampus, L** | **Putamen, R** | **Putamen, L** | **Thalamus, R** | **Thalamus, L** | **Amygdala, R** | **Amygdala, L** |
| --- | --- | --- | --- | --- | --- | --- | --- | --- | --- |
| **Alcohol** | **-0.10^***^ (-0.11, -0.09)** | **-0.04^***^ (-0.05, -0.03)** | **-0.04^***^ (-0.05, -0.03)** | **-0.03^***^ (-0.04, -0.02)** | **-0.03^***^ (-0.04, -0.02)** | **-0.06^***^ (-0.07, -0.05)** | **-0.05^***^ (-0.06, -0.04)** | 8.16x10^-3^ (-5.37x10^-3^ to 2.17x10^-2^) | -8.87x10^-3^ (-2.22x10^-2^ to 4.44x10^-3^) |
| **Age** | 1.07 (-0.33,2.47) | 1.16 (-0.61,2.93) | 0.38 (-1.42,2.17) | 0.78 (-0.78,2.33) | 0.95 (-0.63,2.52) | **1.85^***^ (0.47, 3.23)** | 1.32 (-0.09,2.72) | -0.18(-0.43 to 0.008) | 3.24x10^-2^ (-0.22 to -0.28) |
| **Sex** | **-0.71^***^ (-1.20, -0.23)** | 0.28  (-0.33,0.90) | 0.19 (-0.43,0.81) | 0.16 (-0.38,0.70) | 0.41 (-0.14,0.96) | 0.27 (-0.21,0.75) | 0.26 (-0.23,0.76) | -0.76 (-2.08 to 0.56) | -0.11 (-1.40 to 1.19) |
| **Age^2^** | -2.96^*^  (-5.78,-0.14) | -1.63 (-5.21,1.94) | -0.22 (-3.83,3.40) | -1.72 (-4.86,1.42) | -1.89 (-5.07,1.28) | **-4.01^***^ (-6.79, -1.22)** | -2.92^*^ (-5.77, -0.08) | 3.01x10^-3^ (-1.72x10^-3^ to 7.73x10^-3^) | -5.95x10^-4^ (-5.24x10^-3^ to 4.05x10^-3^) |
| **Age^3^** | 1.28 (-0.16,2.72) | 0.27 (-1.55,2.09) | -0.38 (-2.22,1.46) | 0.69 (-0.91,2.29) | 0.67 (-0.95,2.28) | 1.85^*^ (0.43,3.27) | 1.31 (-0.14,2.76) | -1.72x10^-5^ (-4.61x10^-5^ to 1.17x10^-5)^ | 3.37x10^-6^ (-2.51x10^-5^ to 3.18x10^-5^ |
| **Age x sex** | 1.12^*^ (0.09, 2.14) | -0.09 (-1.39,1.21) | 0.12 (-1.19,1.44) | 0.26 (-0.88,1.40) | -0.34 (-1.49,0.82) | -0.17 (-1.18,0.84) | -0.12 (-1.15,0.91) | 3.63x10^-2^ (-1.29x10^-2^ to 8.54x10^-2^) | 1.46x10^-2^ (-3.37x10^-2^ to 6.30x10^-2^) |
| **Age^2^ x sex** | -0.45 (-1.01,0.11) | -0.18 (-0.89,0.53) | -0.29 (-1.01,0.42) | -0.28 (-0.90,0.34) | 0.08 (-0.55,0.71) | -0.05 (-0.60,0.50) | -0.11 (-0.67,0.45) | -3.29x10^-4^ (-7.81x10^-4^ to 1.23x10^-4^) | -1.50x10^-4^ (-5.94x10^-4^ to 2.94x10^-4^) |
| **Higher degree** | -0.02  (-0.06,0.03) | 0.03 (-0.02,0.09) | 0.02 (-0.03,0.08) | 0.05^*^ (0.002,0.10) | 0.01  (-0.04,0.06) | 0.05^*^ (0.01,0.09) | 0.04 (-0.00,0.09) | 8.22x10^-2^ (2.33x10^-2^ to 0.14) | 8.49x10^-2 **^(2.70x10^-2^ to 0.14) |
| **A levels** | 0.01 (-0.04,0.06) | 0.01 (-0.05,0.07) | 0.01 (-0.05,0.08) | 0.04 (-0.01,0.09) | -0.01 (-0.06,0.05) | 0.05^*^ (0.00,0.10) | 0.03 (-0.01,0.08) | 5.88x10^-2^ (-6.77x10^-3^ to 0.14) | 3.56x10^-2^ (-2.89x10^-2^ to 0.10) |
| **GCSEs** | -0.01 (-0.05,0.04) | -0.01 (-0.07,0.05) | -0.02 (-0.08,0.04) | 0.05^*^ (-0.00,0.10) | 0.001 (-0.05,0.05) | 0.03 (-0.01,0.08) | 0.03 (-0.02,0.07) | 6.02x10^-2^ (-1.93x10^-3^ to 0.12) | 1.31x10^-2^ (-4.80x10^-2^ to 0.07) |
| **CSEs** | 0.03 (-0.03, 0.09) | -0.004 (-0.08, 0.08) | 0.003 (-0.08, 0.08) | 0.03 (-0.04, 0.10) | -0.01 (-0.08, 0.06) | 0.04 (-0.02, 0.10) | 0.02 (-0.05, 0.08) | 8.77x10^-2^ (2.09x10^-3^ to 0.17) | 5.75x10^-2^ (-2.66x10^-2^ to 0.14) |
| **NVQ** | -0.01 (-0.06, 0.05) | -0.003 (-0.07, 0.07) | 0.01 (-0.06, 0.08) | 0.04 (-0.02, 0.10) | 0.00 (-0.07, 0.06) | 0.00 (-0.05, 0.06) | 0.00 (-0.05, 0.06) | 4.26x10^-2^ (-3.42x10^-2^ to 0.12) | 9.27x10^-2^ (1.62x10^-2^ to 0.17) |
| **Professional qualification** | 0.05 (-0.01, 0.11) | 0.01 (-0.07, 0.08) | 0.01 (-0.06, 0.09) | 0.07^*^ (0.01, 0.14) | 0.03 (-0.04, 0.10) | 0.02 (-0.04, 0.08) | 0.02 (-0.04, 0.07) | 3.06x10^-2^ (-4.90x10^-2^ to 0.11) | 6.51x10^-2^ (-1.32x10^-2^  to 0.14) |
| **Site 1** | **-0.15^***^ (-0.18, -0.12)** | **-0.07^***^ (-0.10, -0.04)** | **-0.08^***^ (-0.12, -0.05)** | **-0.12^***^ (-0.15, -0.09)** | **-0.13^***^ (-0.16, -0.10)** | **-0.11^***^ (-0.14, -0.08)** | **-0.11^***^ (-0.13, -0.08)** | **-5.30x10^-2 ***^(-8.93x10^-2^ to -1.67x10^-2^)** | **-4.12x10^-2 ***^(-7.69x10^-2^ to -5.46x10^-3^)** |
| **Site 2** | **-0.12^***^ (-0.14, -0.10)** | **-0.04^***^ (-0.07, -0.01)** | **-0.08^***^ (-0.11, -0.05)** | **-0.08^***^ (-0.10, -0.05)** | **-0.09^***^ (-0.11, -0.06)** | **-0.04^***^ (-0.06, -0.01)** | **-0.05^***^ (-0.07,-0.02)** | **-0.11^***^ (-0.14 to -0.08)** | **-0.13^***^ (-0.16 to -0.10)** |
| **Previous vs. never smoker** | **-0.06^***^ (-0.08, -0.04)** | -0.01 (-0.04, 0.02) | -0.003 (-0.03, 0.02) | -0.01 (-0.04, 0.01) | -0.02 (-0.05, 0.0001) | **-0.04^***^ (-0.06, -0.02)** | **-0.04^***^ (-0.06, -0.02)** | 3.79x10^-3^ (-2.40x10^-2^ to 3.16x10^-2^) | 3.44x10^-3^ (-2.39x10^-2^ to 3.08x10^-2)^ |
| **Current vs. never smoker** | **-0.16^***^ (-0.19, -0.12)** | -0.06^*^ (-0.11, -0.01) | -0.07^*^ (-0.12, -0.02) | -0.02 (-0.06, 0.03) | -0.03 (-0.08, 0.01) | **-0.11^***^ (-0.15, -0.07)** | **-0.10^***^ (-0.14, -0.06)** | -5.17x10^-2^ (-0.11 to 2.01x10^-3^) | -4.33x10^-2^ (-9.61x10^-2^ to 9.48x10^-3^) |
| **TDI** | **-0.02^***^ (-0.03, -0.01)** | -0.01 (-0.02, 0.003) | -0.01^*^ (-0.02, -0.001) | -0.001 (-0.01, 0.01) | -0.0001 (-0.01, 0.01) | -0.01^*^(-0.02,-0.00) | -0.01^*^ (-0.02,0.00) | 1.44x10^-3^ (-3.40x10^-3^ to 6.27x10^-3^) | -1.41x10^-3^ (-6.16x10^-3^ to 3.35x10^-3^) |
| **SBP** | **0.02^***^ (0.01,0.04)** | 0.00(-0.02, 0.02) | 0.00(-0.02,0.02) | 0.02^*^ (0.00,0.04) | 0.01 (-0.01, 0.02) | 0.02^*^(0.00, 0.03) | 0.02^*^ (0.00, 0.03) | -6.04x10^-4^ (-1.63x10^-3^ to 4.28x10^-4)^ | -1.25x10^-4^ (-1.14x10^-3^ to 8.89x10^-4^) |
| **DBP** | **-0.04^***^ (-0.06, -0.03)** | 0.01(-0.01, 0.03) | 0.00(-0.01, 0.02) | -0.01(-0.02, 0.01) | 0.00(-0.02, 0.01) | **-0.02^***^ (-0.04, -0.01)** | **-0.03^***^ (-0.04, -0.01)** | -1.17x10^-4^ (-1.92x10^-3^ to 1.69x10^-3^) | -2.90x10^-4^ (-2.06x10^-3^ to 1.38x10^-3^) |
| **BMI** | **-0.05^***^ (-0.06, -0.04)** | 0.00(-0.02, 0.01) | -0.02^*^(-0.03,0.00) | **-0.04^***^ (-0.05, -0.03)** | **-0.03^***^ (-0.04, -0.02)** | **-0.04^***^ (-0.05, -0.03)** | **-0.04^***^ (-0.05, -0.03)** | 5.81x10^-4^ (-2.82x10^-3^ to 3.98x10^-3^) | 3.10x10^-3^ (-2.51x10^-4^ to 6.44x10^-3^) |
| **MET minutes weekly** | 0.00(-0.01, 0.01) | 0.01 (0.00, 0.02) | 0.01(0.00, 0.02) | 0.01(-0.01, 0.02) | 0.01 (0.00, 0.02) | 0.01(0.00, 0.02) | 0.01(-0.01, 0.01) | 1.86x10^-4 **^(4.59x10^-5^ to 3.26x10^-4^) | 1.06x10^-4^ (-3.21x10^-5^ to 2.43x10^-4^) |
| **Diabetes mellitus** | **-0.27^***^ (-0.31, -0.22)** | **-0.11^***^ (-0.16, -0.05)** | **-0.11^***^ (-0.16, -0.05)** | **-0.12^***^ (-0.17, -0.07)** | **-0.16^***^ (-0.22, -0.11)** | **-0.18^***^ (-0.22, -0.13)** | **-0.19^***^ (-0.23, -0.14)** | 2.89x10^-2^ (-3.19x10^-2^ to 8.97x10^-2^) | -7.94x10^-2^ **(-0.13 to -1.96x10^-2^) |
| **Non-HDL cholesterol** | **0.02^***^ (0.01, 0.03)** | 0.00(-0.01, 0.01) | 0.00(-0.01, 0.02) | 0.00(-0.01, 0.01) | -0.01(-0.02, 0.00) | 0.00(-0.01, 0.01) | 0.00(-0.01, 0.01) | 9.96x10^-3^ (-2.85x10^-3^ to 2.28x10^-2^) | 6.91x10^-3^ (6.96x10^-3^ to 6.43x10^-3^) |
| **Estimated intracranial volume** | **0.30^***^ (0.28, 0.31)** | **-0.37^***^ (-0.38, -0.35)** | **-0.32^***^ (-0.34, -0.31)** | **-0.47^***^ (-0.48, -0.45)** | **-0.45^***^ (-0.46, -0.44)** | **-0.62^***^ (-0.63, -0.61)** | **-0.61^***^ (-0.62, -0.60)** | **-1.84^***^ (-1.97 to -1.70)** | **-2.15^***^ (-2.29 to -2.02)** |
| **N** | 22,254 | 22,254 | 22,254 | 22,254 | 22,254 | 22,254 | 22,254 | 22,254 | 22,254 |
| **R^2^** | 0.51 | 0.21 | 0.19 | 0.39 | 0.38 | 0.52 | 0.50 | 0.10 | 0.13 |
| **Adjusted R^2^** | 0.51 | 0.21 | 0.19 | 0.39 | 0.38 | 0.52 | 0.50 | 0.10 | 0.13 |
| **Residual Std. Error (df = 22228)** | 0.70 | 0.89 | 0.90 | 0.78 | 0.79 | 0.69 | 0.71 | 0.95 | 0.93 |
| **F Statistic (df = 25; 22228)** | 921.30^**^ | 241.09^**^ | 214.87^**^ | 574.86^**^ | 540.86^**^ | 972.97^**^ | 896.36^**^ | 96.02** | 129.8** |

**Table 8: Regression models for total and subcortical grey matter volumes (standardised estimates). Abbreviations: L – left, R – right, TDI – Townsend Deprivation Index, SBP – systolic blood pressure, DBP – diastolic blood pressure, BMI – body mass index, MET – Metabolic Equivalent Task, HDL – high-density lipoproteins, df – degrees of freedom**. **^*^uncorrected p < .05; ^**^** **uncorrected p < .01. ***Bonferroni threshold <0.006.**

**
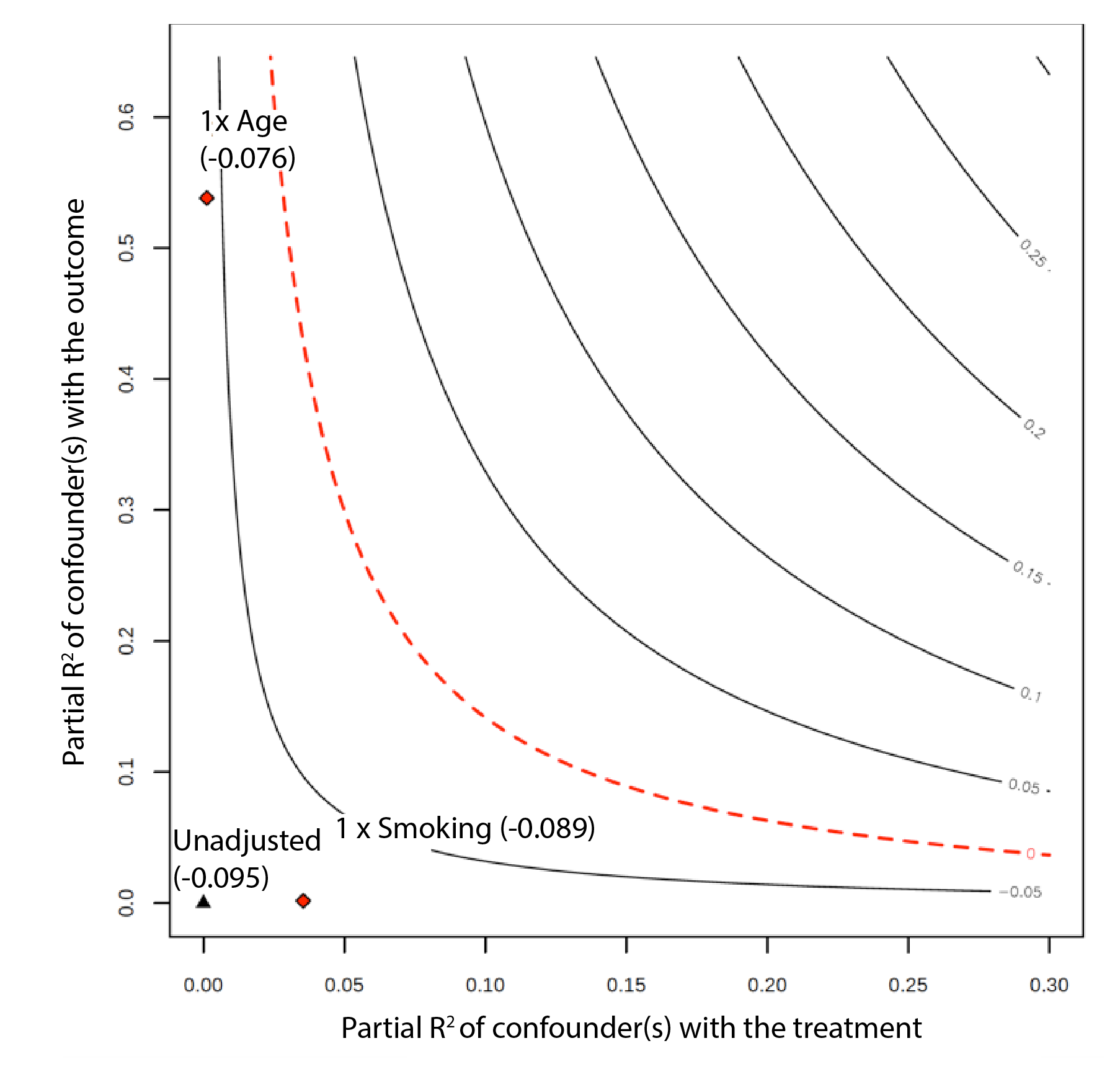
**

**Figure 8: Sensitivity contour plot of the point estimate for the association of alcohol intake and grey matter volume. The unadjusted estimate is shown by the black triangle. Contours indicate how the estimate would change based on the strength of an unobserved confounder with the treatment (alcohol intake – x axis) and outcome (total grey matter volume – y axis). Red diamonds indicate the strength of the observed confounders age and smoking.**

|  | **Digit span** | **Trails A duration** | **Trails B duration** | **Matrix puzzle** | **Tower correlation** | **Fluid intelligence** | **Reaction time** | **Prospective memory** | **Pairs matching** |
| --- | --- | --- | --- | --- | --- | --- | --- | --- | --- |
| **GM (normalised)** | 0.02(0.002, 0.04) | **-0.05^***^ (-0.07, -0.03)** | **-0.05^***^ (-0.07, -0.03)** | **0.04^***^ (0.02, 0.06)** | **0.04^***^ (0.02, 0.06)** | 0.01 (-0.01, 0.03) | **-0.05^***^ (-0.07 to -0.03)** | -2.56e-2(-0.06 to 0.00) | -0.01(-0.03 to 0.01) |
| **Age, *years*** | **-0.10^***^ (-0.12, -0.07)** | **0.25^***^ (0.23, 0.27)** | **0.22^***^ (0.20, 0.24)** | **-0.18^***^ (-0.20, -0.16)** | **-0.17^***^ (-0.19, -0.15)** | **-0.04^***^ (-0.06, -0.02)** | **-0.05^***^ (-0.07 to -0.03)** | **6.1e-3^***^ (0.00 to 0.01)** | **0.01^***^ (0.01 to 0.01)** |
| **Sex** | **0.15^***^ (0.12, 0.19)** | 0.03 (-0.01, 0.06) | -0.03 (-0.06, 0.01) | **0.20^**^ (0.17, 0.24)** | **0.21^***^ (0.18, 0.25)** | **0.16^***^ (0.13, 0.19)** | **0.21^***^ (0.18 to 0.24)** | **9.69e-2^***^ (0.05 to 0.15)** | **0.06^***^ (0.02 to 9.09)** |
| **Higher degree vs. no educational qualification** | **0.67^***^ (0.59, 0.74)** | **-0.46^***^ (-0.53, -0.38)** | **-0.67^***^ (-0.75, -0.60)** | **0.96^***^ (0.89, 1.04)** | **0.53^***^ (0.45, 0.60)** | **1.14^***^ (1.07, 1.22)** | **-0.22^***^ (-0.29 to -0.16)** | -9.45-2(-0.20 to 0.01) | **-0.22^***^ (-0.28 to -0.15)** |
| **A levels vs. no educational qualification** | **0.61^**^ (0.52, 0.69)** | **-0.46^**^ (-0.55, -0.38)** | **-0.63^**^ (-0.71, -0.54)** | **0.79^**^ (0.70, 0.87)** | **0.49^**^ (0.41, 0.58)** | **0.90^**^ (0.82, 0.99)** | **-0.17(-0.25 to -0.10) ^**^** | -1.34e-1(-0.25 to -0.02) | **-0.24(-0.32 to -0.17) ^**^** |
| **GCSEs vs. no educational qualification** | **0.46^***^ (0.37, 0.54)** | **-0.42^***^ (-0.50, -0.34)** | **-0.49^***^ (-0.58, -0.41)** | **0.49^***^ (0.41, 0.57)** | **0.37^***^ (0.29, 0.46)** | **0.60^***^ (0.52, 0.68)** | **-0.12^***^ (-0.19 to -0.05)** | -6.44e-2-0.18 to 0.05) | **-0.18^***^ (-0.25 to -0,11)** |
| **CSEs vs. no educational qualification** | 0.09 (-0.02, 0.20) | **-0.29^***^ (-0.40, -0.18)** | **-0.19^***^ (-0.30, -0.08)** | **0.18^***^ (0.07, 0.28)** | **0.19^***^ (0.08, 0.30)** | 0.08 (-0.03, 0.19) | -0.04(-0.14 to 0.05) | -2.27e-2(-0.17 to 0.13) | -0.05(-0.15 to 0.04) |
| **NVQ vs. no educational qualification** | **0.23^***^ (0.13, 0.33)** | **-0.27^***^ (-0.37, -0.17)** | **-0.31^***^ (-0.41, -0.21)** | **0.39^***^ (0.29, 0.48)** | **0.25^***^ (0.15, 0.35)** | **0.37^***^ (0.27, 0.47)** | -0.09(-0.18 to -0.01) | 3.73e-2(0.10 to 0.18) | -0.07(-0.16 to 0.02) |
| **Professional qualification vs. no educational qualification** | **0.21^***^ (0.11, 0.32)** | **-0.28^***^ (-0.38, -0.17)** | **-0.40^***^ (-0.50, -0.30)** | **0.40^***^ (0.30, 0.50)** | **0.25^***^ (0.15, 0.36)** | **0.51^***^ (0.41, 0.62)** | -0.05(-0.14 to 0.04) | -2.62e-2(0.17 to 0.11) | -0.05-0.14 to 0.04) |
| **Previous vs. never smoker** | 0.05^**^ (0.02, 0.09) | -0.02 (-0.06, 0.01) | 0.02 (-0.02, 0.05) | -0.03 (-0.06, 0.004) | 0.04^*^ (0.004, 0.07) | 0.01 (-0.03, 0.04) | **-0.05^***^ (-0.08 to -0.02)** | -2.26e-2(-0.07 to 0.03) | 0.00(-0.03 to 0.03) |
| **Current vs. never smoker** | 0.08^*^ (0.01, 0.15) | 0.03 (-0.04, 0.10) | 0.05 (-0.02, 0.12) | -0.01 (-0.07, 0.06) | 0.07 (-0.002, 0.13) | -0.01 (-0.07, 0.06) | 0.05(-0.01 to 0.11) | 4.98e-2(-0.05 to 0.15) | 0.02(-0.04 to 0.08) |
| **TDI** | **-0.04^***^ (-0.05, -0.02)** | **0.04^***^ (0.02, 0.05)** | **0.03^***^ (0.02, 0.05)** | -0.04^**^ (-0.05, -0.02) | **-0.04^***^ (-0.05, -0.02)** | **-0.04^***^ (-0.05, -0.02)** | **0.01^***^ (0.00 to 0.01)** | -2.55e-3(-0.01 to 0.01) | 0.01(0.01 to 0.02) |

**Table 9: Regression models predicting cross-sectional cognitive performance at the time of scanning with total grey matter volume as an independent variable (standardised estimates). Abbreviations: TDI – Townsend Deprivation Index, GM – (total) grey matter. ^*^** **uncorrected p < .05; ^**^ uncorrected p < .01. ***Bonferroni threshold <0.006.**

| \|  \| **Reaction time** \| **Prospective memory** \| **Fluid intelligence** \| \| --- \| --- \| --- \| --- \| \| **Alcohol, *weekly units*** \| 0.05 (-0.003, 0.10) \| 0.06 (-0.03, 0.16) \| -0.05 (-0.13, 0.03) \| \| **Higher degree vs. no educational qualification** \| -0.13^**^ (-0.20, -0.06) \| -0.07 (-0.20, 0.05) \| 0.54^**^ (0.44, 0.65) \| \| **A levels vs. no educational qualification** \| -0.07 (-0.15, 0.01) \| -0.08 (-0.22, 0.06) \| 0.44^**^ (0.32, 0.55) \| \| **GCSEs vs. no educational qualification** \| -0.04 (-0.11, 0.03) \| -0.11 (-0.24, 0.03) \| 0.33^**^ (0.22, 0.44) \| \| **CSEs vs. no educational qualification** \| -0.01 (-0.10, 0.09) \| -0.09 (-0.26, 0.09) \| 0.12 (-0.03, 0.26) \| \| **NVQ vs. no educational qualification** \| -0.01 (-0.10, 0.08) \| 0.02 (-0.14, 0.18) \| 0.14^*^ (0.01, 0.28) \| \| **Professional qualification vs. no educational qualification** \| -0.01 (-0.10, 0.08) \| -0.14 (-0.30, 0.02) \| 0.28^**^ (0.14, 0.42) \| \| **Age, *years*** \| 0.18^**^ (0.17, 0.20) \| 0.03^*^ (0.01, 0.05) \| -0.03^*^ (-0.05, -0.005) \| \| **Sex** \| -0.12^**^ (-0.15, -0.09) \| -0.09^**^ (-0.14, -0.04) \| 0.07^**^ (0.03, 0.11) \| \| **TDI** \| 0.02^**^ (0.01, 0.04) \| 0.01 (-0.02, 0.03) \| -0.02^*^ (-0.04, -0.003) \| \| **BMI, *kg/m^2^*** \| 0.004 (-0.01, 0.02) \| 0.001 (-0.02, 0.02) \| -0.003 (-0.02, 0.02) \| \| **Previous vs. never smoker** \| -0.03^*^ (-0.06, -0.001) \| -0.04 (-0.09, 0.01) \| -0.03 (-0.08, 0.01) \| \| **Current vs. never smoker** \| 0.07^*^ (0.01, 0.13) \| -0.05 (-0.16, 0.06) \| -0.05 (-0.14, 0.03) \| \| **Metabolic Equivalent Task, *minutes weekly*** \| -0.01 (-0.02, 0.01) \| -0.01 (-0.03, 0.01) \| -0.03^**^ (-0.05, -0.01) \| \| **Baseline reaction time** \| 0.44^**^ (0.43, 0.46) \|  \|  \| \| **Baseline prospective memory** \|  \| 0.04^**^ (0.01, 0.06) \|  \| \| **Baseline fluid intelligence** \|  \|  \| 0.58^**^ (0.56, 0.60) \| \| **Baseline pairs matching** \|  \|  \|  \| \| **Time between visits** \| 0.08^**^ (0.06, 0.09) \| 0.0002 (-0.03, 0.03) \| -0.06^**^ (-0.08, -0.03) \| \| **Alcohol x Degree** \| -0.06^*^ (-0.12, -0.003) \| -0.02 (-0.12, 0.08) \| 0.06 (-0.02, 0.14) \| \| **Alcohol x A levels^1^** \| -0.06 (-0.12, 0.01) \| -0.07 (-0.19, 0.04) \| 0.07 (-0.03, 0.16) \| \| **Alcohol x GCSEs^1^** \| -0.03 (-0.09, 0.04) \| -0.04 (-0.15, 0.07) \| 0.10^*^ (0.01, 0.19) \| \| **Alcohol x CSEs^1^** \| -0.10^*^ (-0.19, -0.02) \| -0.16^*^ (-0.31, -0.01) \| 0.03 (-0.09, 0.16) \| \| **Alcohol x NVQ^1^** \| -0.06 (-0.13, 0.01) \| -0.06 (-0.20, 0.07) \| 0.01 (-0.10, 0.12) \| \| **Alcohol x Professional qualification^1^** \| -0.04 (-0.12, 0.05) \| -0.05 (-0.20, 0.09) \| 0.06 (-0.06, 0.19) \| \| **Constant** \| 0.15^**^ (0.08, 0.22) \| 0.11 (-0.01, 0.24) \| -0.44^**^ (-0.55, -0.34) \| \| **N** \| 14,506 \| 5,744 \| 5,703 \| \| **R^2^** \| 0.29 \| 0.01 \| 0.43 \| \| **Adjusted R^2^** \| 0.29 \| 0.004 \| 0.43 \| \| **Residual Std. Error** \| 0.84 (df = 14483) \| 0.92 (df = 5721) \| 0.76 (df = 5680) \| \| **F Statistic** \| 265.27^**^ (df = 22; 14483) \| 2.05^**^ (df = 22; 5721) \| 197.96^**^ (df = 22; 5680) \| |
| --- | --- | --- | --- | --- | --- | --- | --- | --- | --- | --- | --- | --- | --- | --- | --- | --- | --- | --- | --- | --- | --- | --- | --- | --- | --- | --- | --- | --- | --- | --- | --- | --- | --- | --- | --- | --- | --- | --- | --- | --- | --- | --- | --- | --- | --- | --- | --- | --- | --- | --- | --- | --- | --- | --- | --- | --- | --- | --- | --- | --- | --- | --- | --- | --- | --- | --- | --- | --- | --- | --- | --- | --- | --- | --- | --- | --- | --- | --- | --- | --- | --- | --- | --- | --- | --- | --- | --- | --- | --- | --- | --- | --- | --- | --- | --- | --- | --- | --- | --- | --- | --- | --- | --- | --- | --- | --- | --- | --- | --- | --- | --- | --- | --- | --- | --- | --- | --- | --- | --- | --- | --- | --- | --- | --- | --- | --- | --- | --- |

**Table 10: Regression models predicting cross-sectional cognitive performance at time of scanning, controlling for baseline performance and alcohol as an independent variable (standardised estimates). ^*^ uncorrected p < .05; ^**^ uncorrected p < .01. Bonferroni threshold <0.003. Only tests with significant alcohol*educational qualification interactions are shown. Abbreviations: GSCEs - General Certificate of Secondary Education, CSEs – Certificate of Secondary Education, NVQ – National Vocational Qualification, TDI – Townsend Deprivation Index, BMI – body mass index.**

**Diffusion tensor imaging analyses**

**
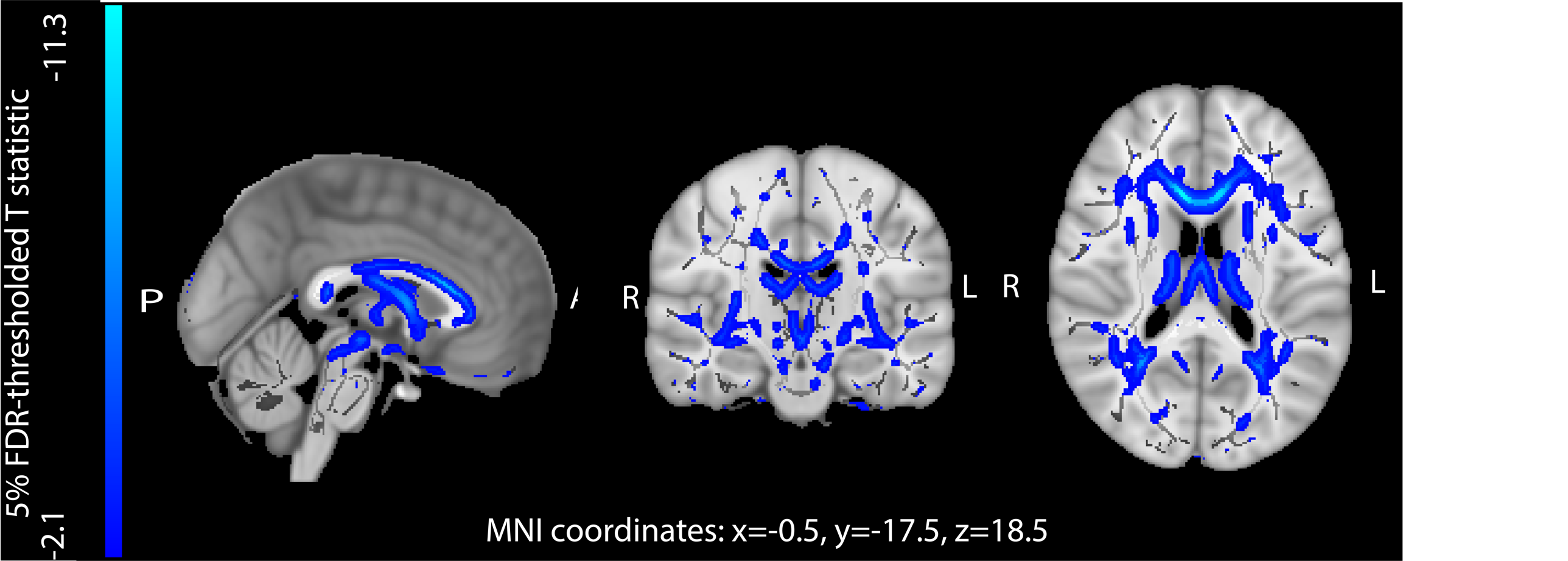
**

**Figure 10: Negative association between alcohol consumption (weekly units) and fractional anisotropy with additional adjustment for image-related confounders. N=22,263. T statistics are thresholded at 5% False Discovery Rate (0.018 threshold on uncorrected p values). Mean FA skeleton shown in grey. Adjusted for: age, sex, age^2^, age^3^, age x sex, age^2^ x sex, imaging site, estimated intracranial size, blood pressure, cholesterol, high-density lipoprotein, Diabetes Mellitus, smoking, body mass index, Metabolic Equivalent Task minutes weekly, Townsend Deprivation Index, head motion, table position, scanner acquisition parameters.**

**
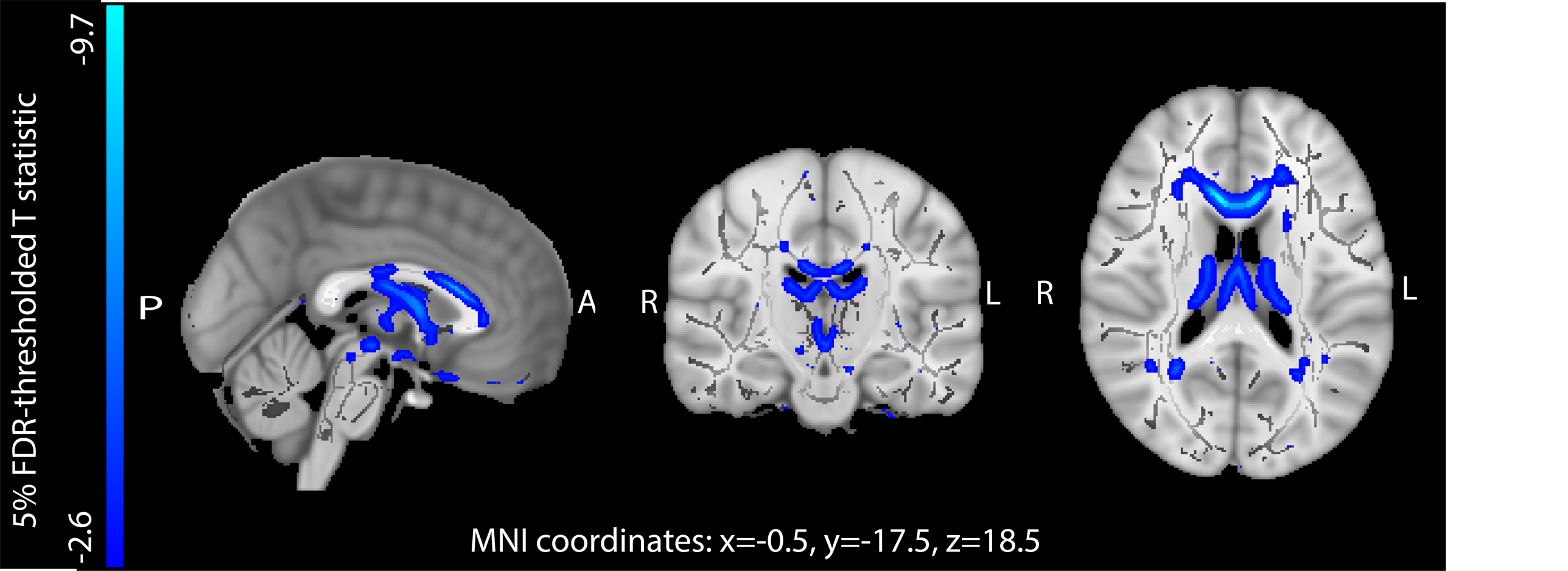
**

**Figure 11: Negative association between alcohol consumption (weekly units) and fractional anisotropy with additional adjustment for global mean fractional anisotropy. N=22,263. T statistics are thresholded at 5% False Discovery Rate (0.004 threshold on uncorrected p values). Mean FA skeleton shown in grey. Adjusted for: age, sex, age^2^, age^3^, age x sex, age^2^ x sex, imaging site, estimated intracranial size, blood pressure, cholesterol, high-density lipoprotein, diabetes mellitus, smoking, body mass index, Metabolic Equivalent Task minutes weekly, Townsend Deprivation Index.**

**
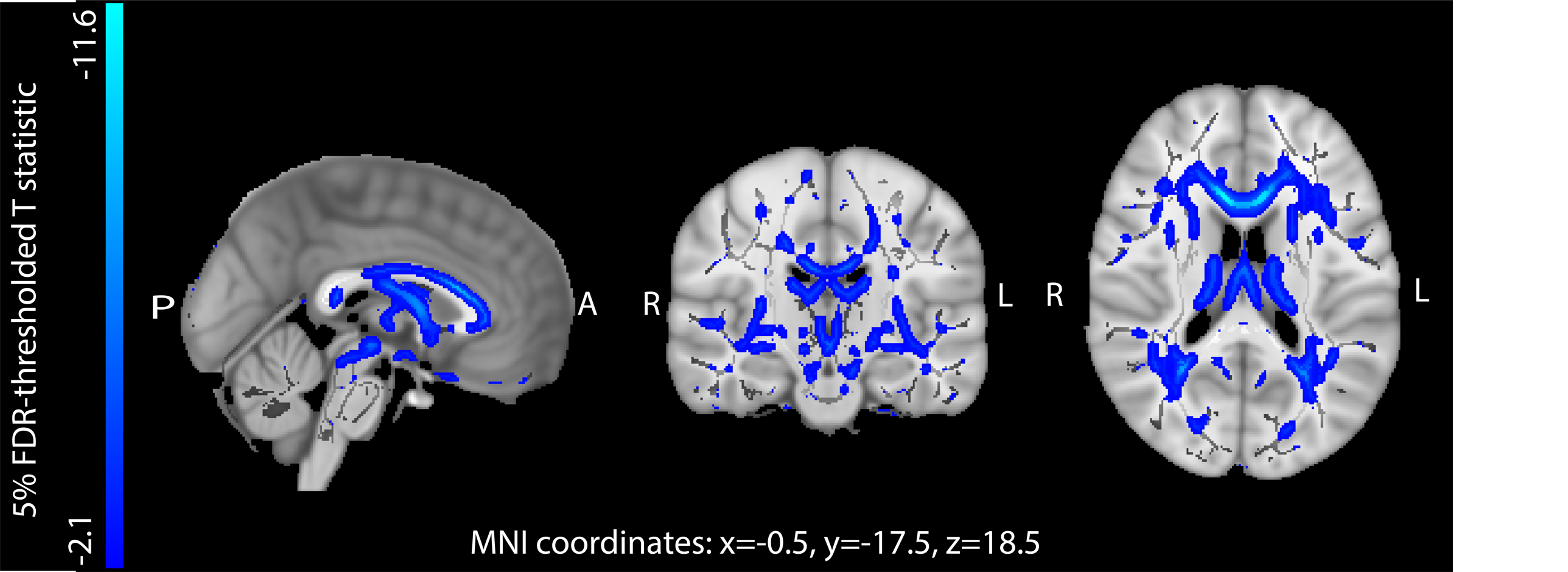
**

**Figure 12: Negative associations between fractional anisotropy and alcohol intake for current drinkers only (n=22,809). T statistics are thresholded at 5% False Discovery Rate (0.019 threshold on uncorrected p values). Mean FA skeleton shown in grey. Adjusted for: age, sex, age^2^, age^3^, age x sex, age^2^ x sex, imaging site, estimated intracranial size, blood pressure, cholesterol, high-density lipoprotein, Diabetes mellitus, smoking, body mass index, Metabolic Equivalent Task minutes weekly, Townsend Deprivation Index.**

**
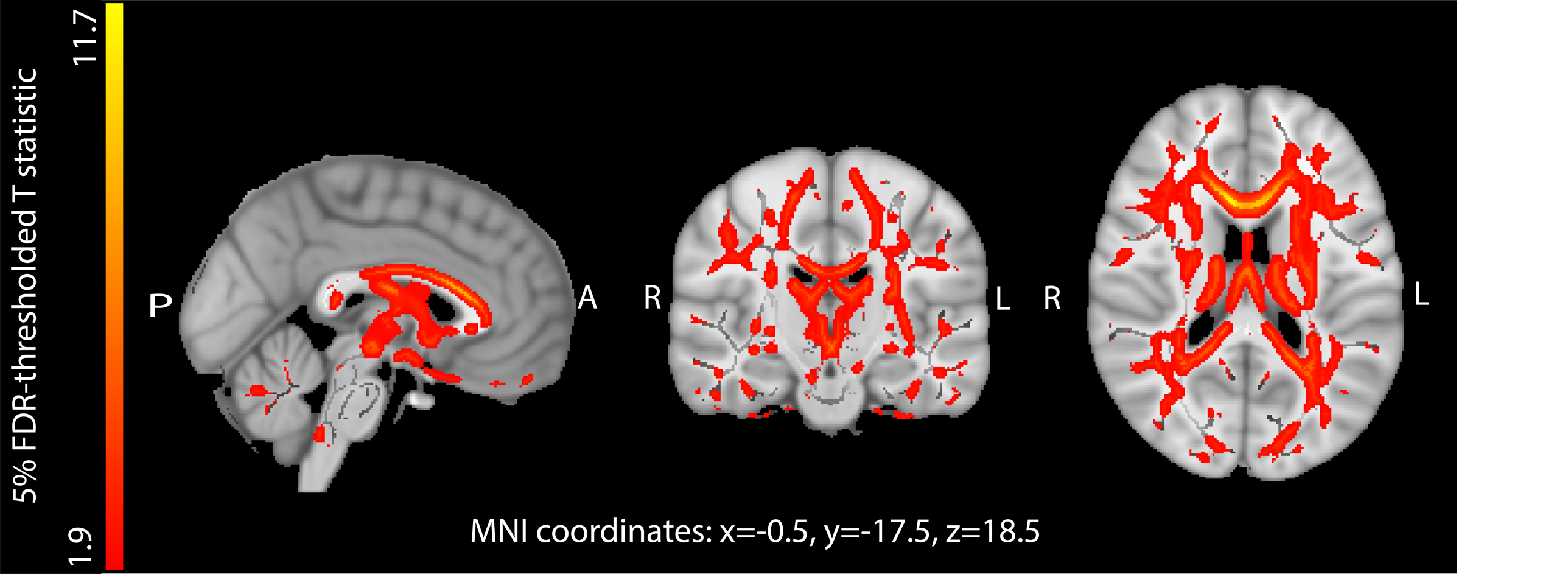
**

**Figure 13: Positive association between alcohol consumption and mean diffusivity. N=24,030 subjects. T statistics are thresholded at 5% False Discovery Rate (0.029 threshold on uncorrected p values). Mean FA skeleton shown in grey. Adjusted for: age, sex, age^2^, age^3^, age x sex, age^2^ x sex, imaging site, estimated intracranial size, blood pressure, cholesterol, high-density lipoprotein, diabetes mellitus, smoking, body mass index, Metabolic Equivalent Task minutes weekly, Townsend Deprivation Index.**

**
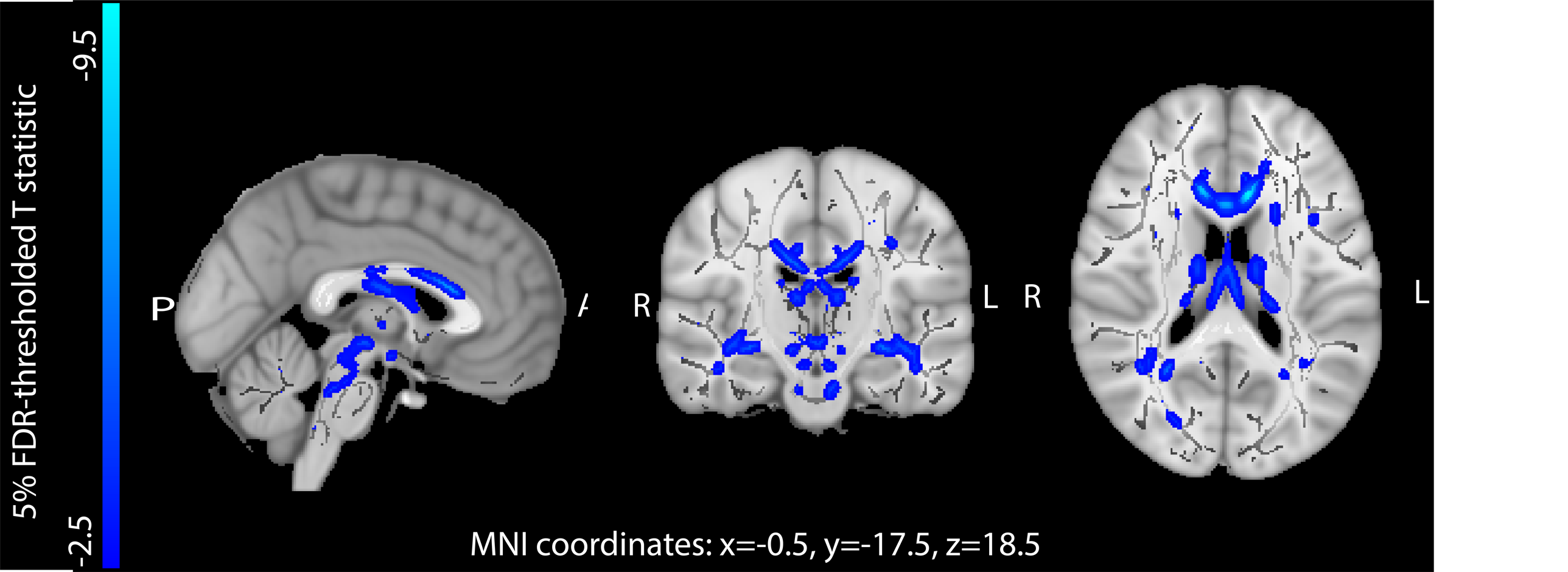
**

**Figure 14: Negative association between alcohol consumption and mode. N=24,030 subjects. T statistics are thresholded at 5% False Discovery Rate (0.006 threshold on uncorrected p values). Mean FA skeleton shown in grey. Adjusted for: age, sex, age^2^, age^3^, age x sex, age^2^ x sex, imaging site, estimated intracranial size, blood pressure, cholesterol, high-density lipoprotein, diabetes mellitus, smoking, body mass index, Metabolic Equivalent Task minutes weekly, Townsend Deprivation Index.**

**
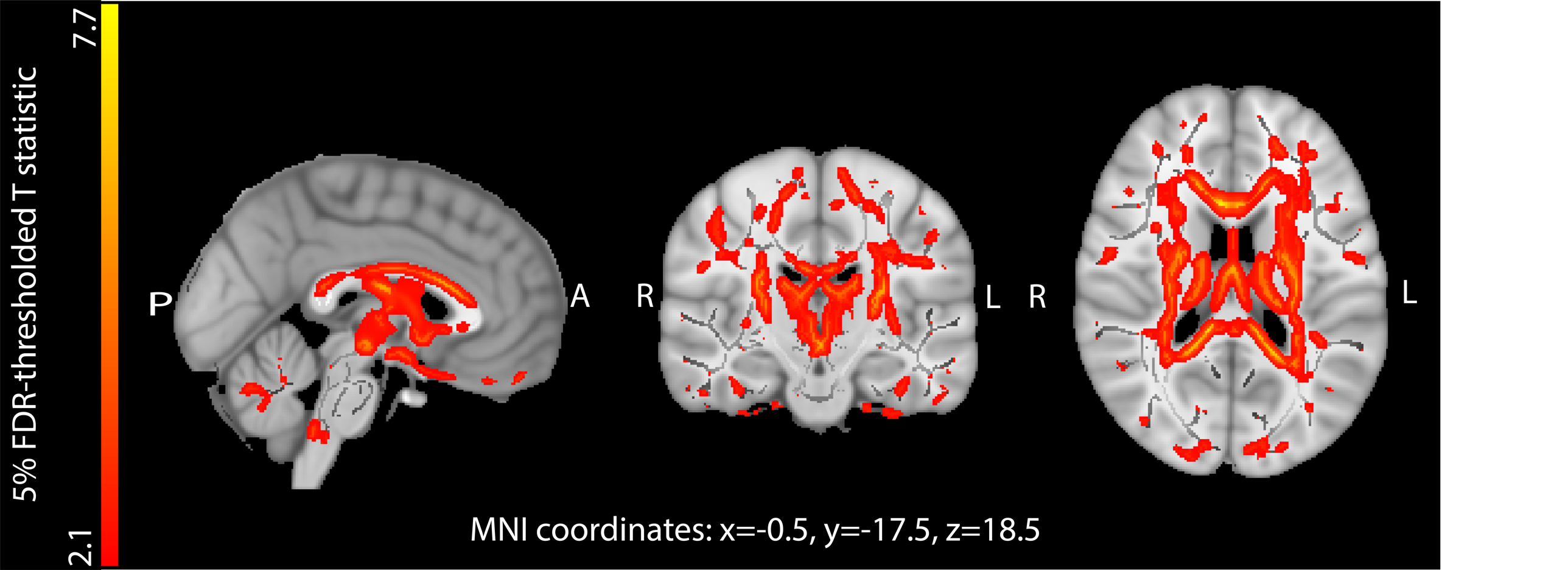
**

**Figure 15: Positive association between alcohol consumption and L1. N=24,030. T statistics are thresholded at 5% False Discovery Rate (0.017 threshold on uncorrected p values). Mean FA skeleton shown in grey. Adjusted for: age, sex, age^2^, age^3^, age x sex, age^2^ x sex, imaging site, estimated intracranial size, blood pressure, cholesterol, high-density lipoprotein, diabetes mellitus, smoking, body mass index, Metabolic Equivalent Task minutes weekly, Townsend Deprivation Index.**

**
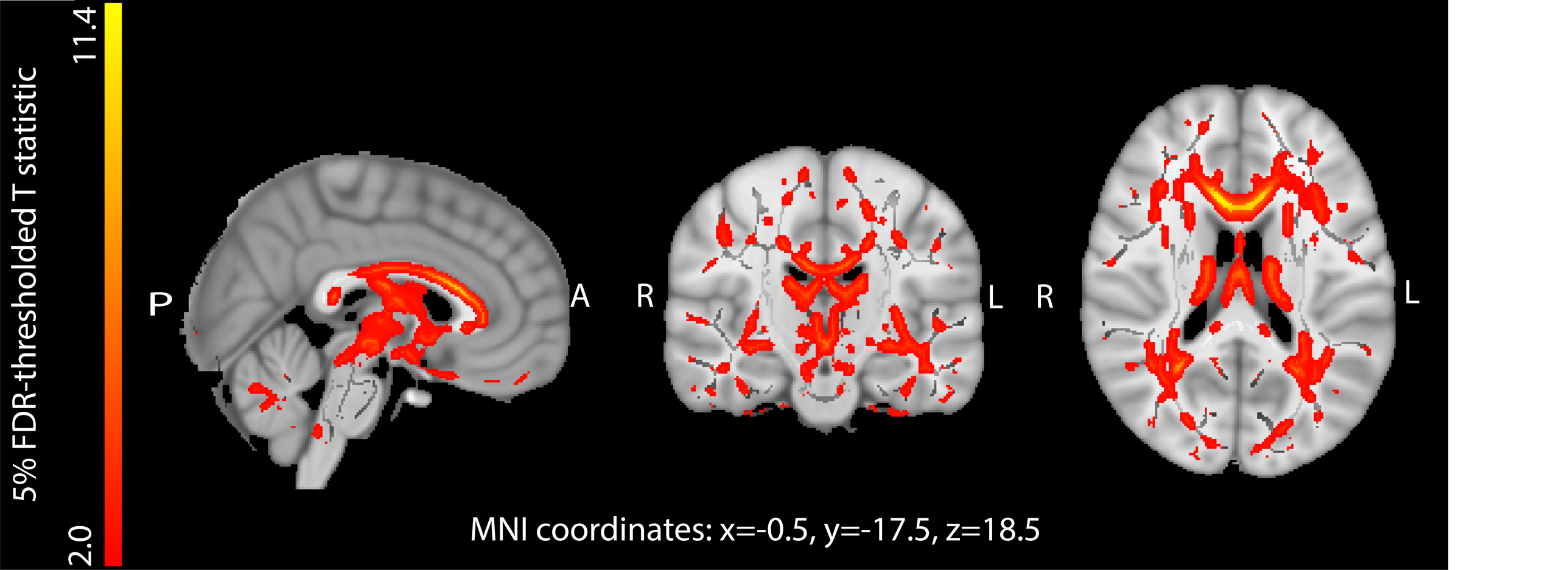
**

**Figure 16: Positive association between alcohol consumption and L2 (radial diffusivity). N=24,030. T statistics are thresholded at 5% False Discovery Rate (0.024 threshold on uncorrected p values). Mean FA skeleton shown in grey. Adjusted for: age, sex, age^2^, age^3^, age x sex, age^2^ x sex, imaging site, estimated intracranial size, blood pressure, cholesterol, high-density lipoprotein, diabetes mellitus, smoking, body mass index, Metabolic Equivalent Task minutes weekly, Townsend Deprivation Index.**

**
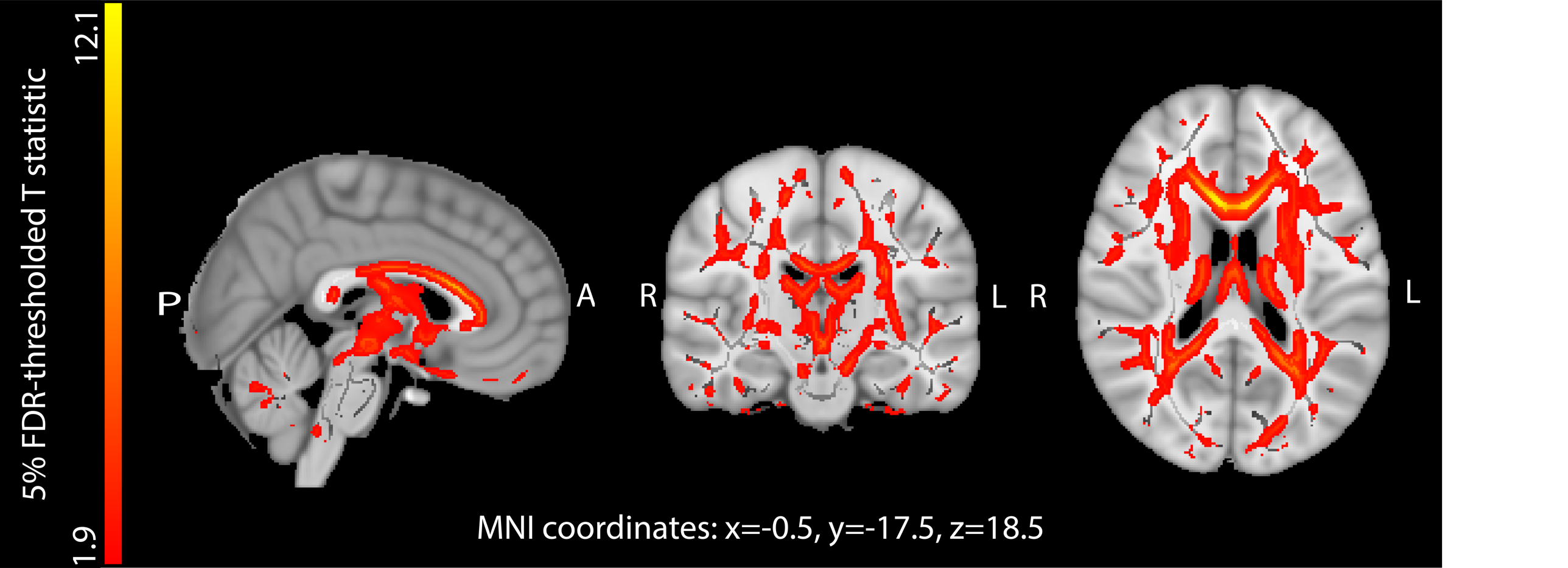
**

**Figure 17: Positive association between alcohol consumption and L3 (radial diffusivity). N=24,030. T statistics are thresholded at 5% False Discovery Rate (0.028 threshold on uncorrected p values). Mean FA skeleton shown in grey. Adjusted for: age, sex, age^2^, age^3^, age x sex, age^2^ x sex, imaging site, estimated intracranial size, blood pressure, cholesterol, high-density lipoprotein, diabetes mellitus, smoking, body mass index, Metabolic Equivalent Task minutes weekly, Townsend Deprivation Index.**

**Functional connectivity analyses**

| **Resting state network** | **Constituent components** |
| --- | --- |
| Default mode | Posterior cingulate, precuneus, medial prefrontal cortex, angular gyrus, hippocampus, temporoparietal junction, lateral temporal cortex |
| Attention | Temporoparietal junction, ventral frontal cortex, intraparietal sulcus, frontal eye fields |
| Salience | Anterior insula, anterior cingulate |
| Central executive | Rostral lateral and dorsolateral prefrontal cortex, inferior parietal lobule, cingulate gyrus, precuneus, temporal lobe, caudate nucleus |
| Visual | Precentral cortex, occipital cortex, angular gyrus |

**Table 11: Key resting state networks and commonly described constituent components** [1-3]**.**

| **Node** | **Regions included** | **Network(s)** |
| --- | --- | --- |
| **3** | Supramarginal, inferior parietal, postcentral | Attention, salience, central executive |
| **4** | Calcarine, lingual, cuneus | Visual |
| **5** | Inferior parietal, cerebellum, angular [primarily right-sided]. | Central executive, attention, default mode |
| **6** | Inferior frontal, middle frontal [primarily left-sided], medial prefrontal, posterior cingulate. | Default mode, central executive |
| **9** | Inferior parietal, angular, middle temporal | Default mode, central executive |
| **13** | Supplementary motor area, middle temporal, inferior frontal | Default mode, salience |
| **21** | Inferior frontal, superior frontal, middle temporal | Default mode, central executive |

**Table 12: Resting state fMRI nodes defined by independent components analysis of 4100 subjects’ images which were significantly associated with alcohol intake. To visualize 3D versions of the group average spatial maps see:** [**https://www.fmrib.ox.ac.uk/ukbiobank/group_means/rfMRI_ICA_d25_good_nodes.html**](https://www.fmrib.ox.ac.uk/ukbiobank/group_means/rfMRI_ICA_d25_good_nodes.html)**)**


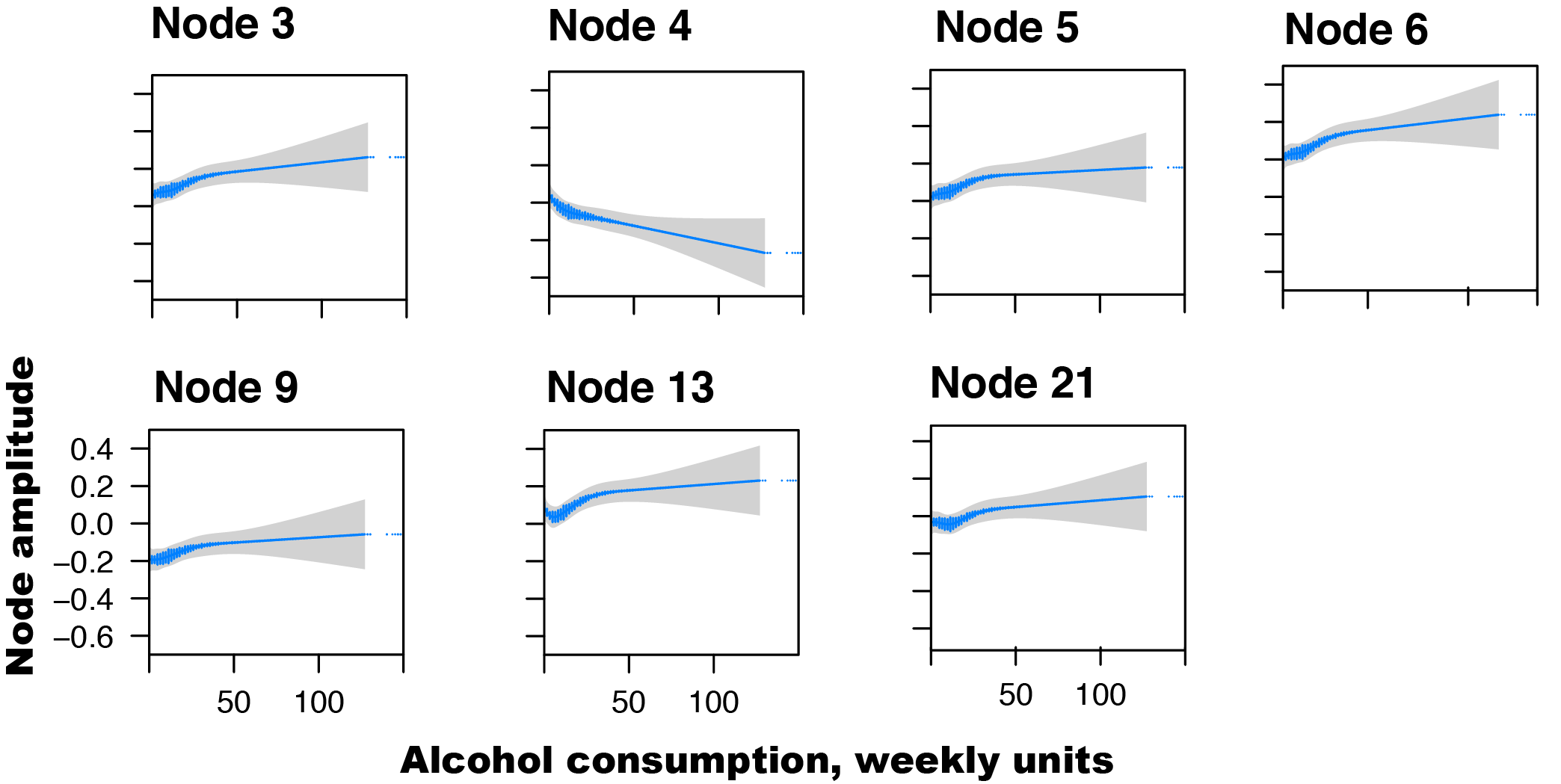


**Figure 18: Associations between alcohol intake weekly and within resting state network connectivity (node amplitude, standardized). Only significant (defined by Bonferroni corrected p<0.002) associations are shown. Splines were fitted (5 knots) to model alcohol intake flexibly. Models adjusted for: age, sex, age^2^, age^3^, age*sex, age^2^*sex, blood pressure, diabetes mellitus, smoking status, Townsend Deprivation Index, body mass index, total cholesterol, high-density lipoprotein, imaging site, head motion and Metabolic Equivalent Task minutes weekly. 95% CI are shaded. N=17,587.**

| Edge | **Nodes connected** | **Regions** | **Networks** |
| --- | --- | --- | --- |
| **145** | 9-18 | Inferior parietal, angular, middle temporal ⬄ putamen/caudate/SMA | DMN/CEN ⬄ subcortical-cerebellum |
| **25** | 4-8 | Calcarine/lingual/cuneus ⬄ lingual/calcarine/superior occipital | Visual ⬄ visual |
| **150** | 14-18 | Anterior cingulate/superior frontal ⬄putamen/caudate/SMA | DMN/salience/CEN ⬄ subcortical-cerebellum |
| **55** | 10-11 | Postcentral/precentral ⬄ postcentral/precentral | Motor ⬄ motor |
| **195** | 5-21 | Inferior parietal/cerebellum/angular ⬄ inferior frontal/superior frontal/middle temporal | CEN/attention/DMN ⬄ DMN/CEN |

**Table 13: Functional connectivity within resting state networks (‘edges’) associated with alcohol intake. Top five most significantly associated edges (as defined by p values) with alcohol intake, together with the regions and networks of their constituent nodes. Abbreviations: SMA – supplementary motor area, DMN – default mode network, CEN – central executive network.**

**
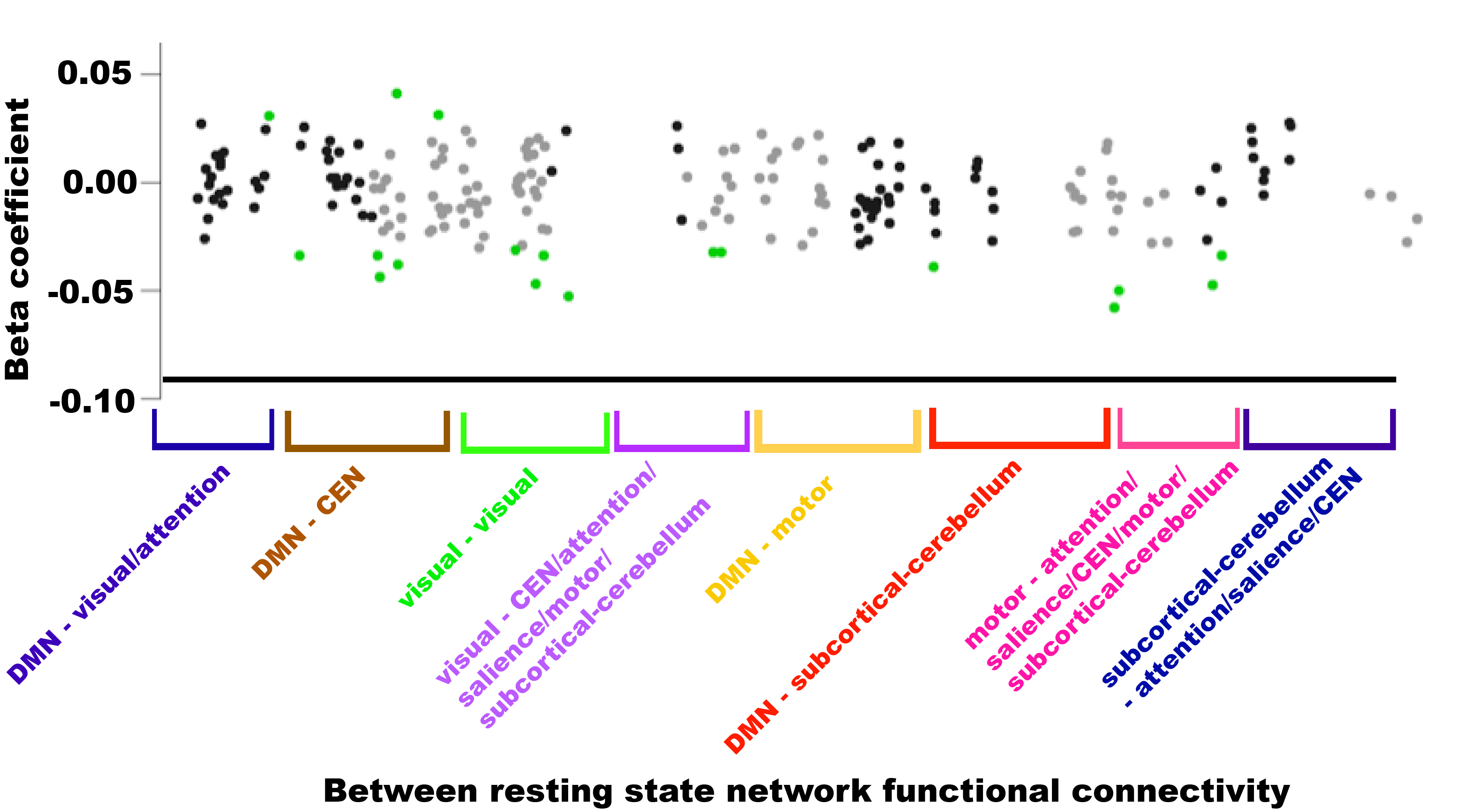
**

**Figure 19: Effect sizes for associations between alcohol consumption and between resting state network connectivity (‘edges’). Manhattan plot shows beta coefficients grouped by networks. Green coloured dots signify Bonferroni significant associations (p<0.0002). Beta coefficients were generated using regression models with functional connectivity (‘edge’) as the dependent variable and alcohol intake as an independent variable, adjusted for: age, sex, age*sex, age^2^, age^2^*sex, blood pressure, diabetes mellitus, smoking status, Townsend Deprivation Index, body mass index, total cholesterol, high-density lipoprotein, imaging site, head motion and Metabolic Equivalent Task minutes weekly. Abbreviations: DMN – default mode network, CEN – central executive network.**

**

**

**Figure 20: Heatmap showing estimates for associations between within resting state functional connectivity (‘nodes’) and cognitive tests performed at the imaging visit. N=7086 subjects included. Estimates (standardized regression coefficients) were generated from regression models adjusted for: age, sex, age*sex, age^2^, age^2^*sex, blood pressure, diabetes mellitus, smoking status, Townsend Deprivation Index, body mass index, total cholesterol, high-density lipoprotein, imaging site, head motion, educational qualifications and Metabolic Equivalent Task minutes weekly. * 5% False Discovery Rate significant (p< 0.01), ** Bonferroni significant (p<0.0008).**

**

**

**Figure 21: Heatmap showing estimates for associations between within resting state functional connectivity (‘nodes’) and cognitive tests performed at the imaging visit controlling for alcohol intake weekly. N=7086 subjects included. Estimates (standardized regression coefficients) were generated from regression models adjusted for: age, sex, age*sex, age^2^, age^2^*sex, blood pressure, diabetes mellitus, smoking status, Townsend Deprivation Index, body mass index, total cholesterol, high-density lipoprotein, imaging site, head motion, educational qualifications and Metabolic Equivalent Task minutes weekly. * 5% False Discovery Rate significant (p< 0.004), ** Bonferroni significant (p<0.0008).**

**Pre-specified subgroup analyses**

|  | | | |
| --- | --- | --- | --- |
|  | **Alcohol-BMI interaction** | **Alcohol-SBP interaction** | **Alcohol-DBP interaction** |
|  | | | |
| **Alcohol, *units weekly*** | **-0.09^***^ (-0.10, -0.08)** | **-0.09^***^ (-0.10, -0.08)** | **-0.09^***^ (-0.10, -0.08)** |
| **Age, *years*** | -0.05^**^ (-0.06, -0.04) | -0.05^**^ (-0.06, -0.04) | -0.05^**^ (-0.06, -0.04) |
| **Sex** | 1.08 (-0.32, 2.48) | 1.03 (-0.37, 2.43) | 1.06 (-0.34, 2.46) |
| **Age^2^** | -0.73^**^ (-1.21, -0.24) | -0.72^**^ (-1.21, -0.24) | -0.74^**^ (-1.23, -0.25) |
| **Age^3^** | -2.99^*^ (-5.81, -0.16) | -2.89^*^ (-5.71, -0.07) | -2.95^*^ (-5.78, -0.13) |
| **Age x sex** | 1.30 (-0.14, 2.73) | 1.25 (-0.19, 2.68) | 1.28 (-0.15, 2.72) |
| **Age^2^ x sex** | 1.14^*^ (0.11, 2.17) | 1.13^*^ (0.10, 2.16) | 1.16^*^ (0.14, 2.19) |
| **Higher degree vs. no educational qualification** | -0.46 (-1.02, 0.10) | -0.45 (-1.01, 0.11) | -0.47 (-1.03, 0.09) |
| **A levels vs. no educational qualification** | -0.02 (-0.06, 0.02) | -0.02 (-0.06, 0.03) | -0.02 (-0.06, 0.03) |
| **GCSEs vs. no educational qualification** | 0.01 (-0.04, 0.06) | 0.01 (-0.04, 0.06) | 0.01 (-0.04, 0.06) |
| **CSEs vs. no educational qualification** | -0.01 (-0.05, 0.04) | -0.01 (-0.05, 0.04) | -0.01 (-0.05, 0.04) |
| **NVQ vs. no educational qualification** | 0.03 (-0.03, 0.09) | 0.03 (-0.03, 0.10) | 0.03 (-0.03, 0.09) |
| **Professional qualification vs. no educational qualification** | -0.01 (-0.06, 0.05) | -0.01 (-0.06, 0.05) | -0.01 (-0.06, 0.05) |
| **Site 1** | 0.05 (-0.01, 0.11) | 0.05 (-0.005, 0.11) | 0.05 (-0.01, 0.11) |
| **Site 2** | **-0.15^***^ (-0.18, -0.12)** | **-0.15^***^ (-0.18, -0.12)** | **-0.15^***^ (-0.18, -0.12)** |
| **Previous vs. never smoker** | **-0.12^***^ (-0.14, -0.10)** | **-0.12^***^ (-0.14, -0.10)** | **-0.12^***^ (-0.14, -0.10)** |
| **Current vs. never smoker** | **-0.06^***^ (-0.08, -0.04)** | **-0.06^***^ (-0.08, -0.04)** | **-0.06^***^ (-0.08, -0.04)** |
| **TDI** | **-0.16^***^ (-0.20, -0.12)** | **-0.16^***^ (-0.20, -0.12)** | **-0.16^***^ (-0.20, -0.12)** |
| **SBP, *mmHg*** | **-0.02^***^ (-0.03, -0.01)** | **-0.02^***^ (-0.03, -0.01)** | **-0.02^***^ (-0.03, -0.01)** |
| **DBP, *mmHg*** | **0.02^***^ (0.01, 0.04)** | **0.02^***^ (0.01, 0.04)** | **0.02^***^ (0.01, 0.04)** |
| **BMI, kg/m^2^** | **-0.04^***^ (-0.06, -0.03)** | **-0.05^***^ (-0.06, -0.03)** | **-0.04^***^ (-0.06, -0.03)** |
| **Metabolic Equivalent Task, *minutes weekly*** | 0.002 (-0.01, 0.01) | 0.002 (-0.01, 0.01) | 0.002 (-0.01, 0.01) |
| **Diabetes mellitus** | **-0.27^***^ (-0.31, -0.22)** | **-0.27^***^ (-0.31, -0.22)** | **-0.27^***^ (-0.31, -0.22)** |
| **Non-HDL cholesterol, *mmol/L*** | **0.02^***^ (0.01, 0.03)** | **0.02^***^ (0.01, 0.03)** | **0.02^***^ (0.01, 0.03)** |
| **Estimated intracranial volume** | **0.30^***^ (0.28, 0.31)** | **0.30^***^ (0.28, 0.31)** | **0.30^***^ (0.28, 0.31)** |
| **Alcohol x BMI** | -0.01^*^ (-0.02, -0.002) |  |  |
| **Alcohol x SBP** |  | **-0.01^***^ (-0.02, -0.004)** |  |
| **Alcohol x DBP** |  |  | **-0.01^***^ (-0.02, -0.004)** |
| **Constant** | 0.10^**^ (0.06, 0.14) | 0.10^**^ (0.06, 0.14) | 0.10^**^ (0.06, 0.14) |
| **N** | 22,254 | 22,254 | 22,254 |
| **R^2^** | 0.51 | 0.51 | 0.51 |
| **Adjusted R^2^** | 0.51 | 0.51 | 0.51 |
| **Residual Std. Error (df = 22227)** | 0.70 | 0.70 | 0.70 |
| **F Statistic (df = 26; 22227)** | 886.24^**^ | 886.44^**^ | 886.42^**^ |
|  | | | |

**Table 14: Regression models for significant interactions with alcohol intake in predicting total grey matter volume (standardised estimates). ^*^ uncorrected p < .05; ^**^ uncorrected p < .01; ^***^Bonferroni threshold p=0.008 (across 6 interactions tested). Abbreviations: BMI – body mass index, SBP – systolic blood pressure, DBP – diastolic blood pressure, TDI – Townsend Deprivation Index, HDL – high-density lipoprotein.**

|  | | |
| --- | --- | --- |
|  | **Alcohol x systolic BP** | **Alcohol x diastolic BP** |
|  | | |
| **Alcohol, *units weekly*** | **-0.09^***^ (-0.10, -0.08)** | **-0.09^***^ (-0.10, -0.08)** |
| **Age, *years*** | 0.90 (-0.50, 2.30) | 0.93 (-0.47, 2.33) |
| **Sex** | **-0.74^***^ (-1.23, -0.25)** | **-0.75^***^ (-1.24, -0.27)** |
| **Age^2^** | -2.63 (-5.45, 0.20) | -2.69 (-5.51, 0.14) |
| **Age^3^** | 1.12 (-0.32, 2.56) | 1.15 (-0.29, 2.59) |
| **Age x sex** | 1.15^*^ (0.12, 2.18) | 1.18^*^ (0.16, 2.21) |
| **Age^2^ x sex** | -0.46 (-1.02, 0.10) | -0.48 (-1.04, 0.08) |
| **Higher degree vs. no educational qualification** | -0.02 (-0.06, 0.03) | -0.02 (-0.06, 0.03) |
| **A levels vs. no educational qualification** | 0.01 (-0.04, 0.05) | 0.01 (-0.04, 0.05) |
| **GCSEs vs. no educational qualification** | -0.01 (-0.05, 0.04) | -0.01 (-0.05, 0.04) |
| **CSEs vs. no educational qualification** | 0.03 (-0.03, 0.10) | 0.03 (-0.03, 0.10) |
| **NVQ vs. no educational qualification** | 0.00 (-0.06, 0.05) | 0.00 (-0.06, 0.05) |
| **Professional qualification vs. none** | 0.05 (-0.01, 0.11) | 0.05 (-0.01, 0.11) |
| **Site 1** | **-0.15^***^ (-0.17, -0.12)** | **-0.15^***^ (-0.17, -0.12)** |
| **Site 2** | **-0.12^***^ (-0.14, -0.10)** | **-0.12^***^ (-0.14, -0.10)** |
| **Previous vs. never smoker** | **-0.06^***^ (-0.08, -0.04)** | **-0.06^***^ (-0.08, -0.04)** |
| **Current vs. never smoker** | **-0.16^***^ (-0.20, -0.12)** | **-0.16^***^ (-0.20, -0.12)** |
| **TDI** | **-0.02^***^ (-0.03, -0.01)** | **-0.02^***^ (-0.03, -0.01)** |
| **SBP, *mmHg*** | **0.03^***^ (0.02, 0.04)** | **0.03^***^ (0.02, 0.04)** |
| **DBP, *mmHg*** | **-0.04^***^ (-0.05, -0.02)** | **-0.04^***^ (-0.05, -0.02)** |
| **BMI, *kg/m^2^*** | **-0.06^***^ (-0.07, -0.05)** | **-0.06^***^ (-0.07, -0.05)** |
| **Metabolic Equivalent Task, *minutes weekly*** | 0.00 (-0.01, 0.01) | 0.00 (-0.01, 0.01) |
| **Diabetes mellitus** | -0.01 (-0.05, 0.04) | -0.01 (-0.05, 0.04) |
| **Non-HDL cholesterol, *mmHg*** | **0.02^***^ (0.01, 0.03)** | **0.02^***^ (0.01, 0.03)** |
| **Estimated intracranial volume** | **0.29^***^ (0.28, 0.31)** | **0.29^***^ (0.28, 0.31)** |
| **BP medication** | -0.11^**^ (-0.14, -0.09) | -0.11^**^ (-0.14, -0.09) |
| **Alcohol x SBP** | **-0.01^***^ (-0.02, 0.00)** |  |
| **Alcohol x DBP** |  | **-0.01^***^ (-0.02, 0.00)** |
| **Constant** | 0.12^**^ (0.07, 0.16) | 0.12^**^ (0.07, 0.16) |
| **N** | 22,254 | 22,254 |
| **R^2^** | 0.51 | 0.51 |
| **Adjusted R^2^** | 0.51 | 0.51 |
| **Residual Std. Error (df = 22226)** | 0.70 | 0.70 |
| **F Statistic (df = 27; 22226)** | 849.56^**^ | 849.54^**^ |
|  | | |
|  | | |

**Table 15: Alcohol-blood pressure interactions, adjusted for self-reported antihypertensive medication, predicting total grey matter volume. Abbreviations: TDI – Townsend Deprivation Index, SBP – systolic blood pressure, DBP – diastolic blood pressure, BMI – body mass index, HDL – high-density lipoprotein. ^*^ uncorrected p < .05; ^**^ uncorrected p < .01; ^***^ Bonferroni p <0.008.**

|  | **Alcohol x age** | **Alcohol x sex** | **Alcohol x Apoe4** |
| --- | --- | --- | --- |
| **Alcohol, *units weekly*** | -0.10^**^ (-0.10 to -0.09) | -0.09^**^ (-0.11 to -0.08) | -0.10^**^ (-0.11 to -0.09) |
| **ApoE4** |  |  | -0.02 (-0.04 to 0.004) |
| **ApoE4/E4** |  |  | 0.04 (-0.02 to -0.10) |
| **Age, *years*** | 1.10 (-0.31 to 2.51) | 1.15 (-0.26 to 2.56) | 1.15 (-0.26 to 2.55) |
| **Sex** | -0.71^**^ (-1.20 to -0.22) | -0.70^**^ (-1.19 to -0.21) | -0.70^**^ (-1.19 to -0.21) |
| **Age^2^** | -3.03^*^ (-5.86 to -0.19) | -3.13^*^ (-5.96 to -0.29) | -3.12^*^ (-5.95 to -0.28) |
| **Age^3^** | 1.32(-0.13 to 2.76) | 1.37 (-0.08 to 2.81) | 1.36 (-0.08 to 2.80) |
| **Age x sex** | 1.08^*^ (0.05 to 2.11) | 1.09^*^ (0.06 to 2.12) | 1.08^*^ (0.05 to 2.11) |
| **Age^2^ x sex** | -0.42 (-0.98 to 0.15) | -0.43 (-1.00 to 0.13) | -0.43(-0.99 to 0.14) |
| **Higher degree vs. no educational qualification** | -0.02 (-0.06 to 0.03) | -0.02 (-0.06 to 0.03) | -0.02 (-0.06 to 0.03) |
| **A levels vs. no educational qualification** | 0.01 (-0.04 to 0.06) | 0.09 (-0.04 to 0.06) | 0.01 (-0.04 to 0.06) |
| **GCSEs vs. no educational qualification** | 0.00 (-0.05 to 0.04) | 0.00 (-0.05 to 0.04) | -0.01 (-0.05 to 0.04) |
| **CSEs vs. no educational qualification** | 0.03 (-0.03 to 0.10) | 0.03 (-0.03 to 0.10) | 0.03 (-0.03 to 0.10) |
| **NVQ vs. no educational qualification** | 0.00 (-0.06 to 0.05) | 0.00 (-0.06 to 0.05) | 0.00 (-0.06 to 0.06) |
| **Professional qualification vs. no educational qualification** | 0.06 (0.00 to 0.11) | 0.06 (0.00 to 0.11) | 0.06 (0.00 to 0.11) |
| **Site 1** | -0.15^**^ (-0.18 to -0.12) | -0.15^**^ (-0.18 to -0.12) | -0.15^**^ (-0.18 to -0.12) |
| **Site 2** | -0.12^**^ (-0.14 o -0.09) | -0.12^**^ (-0.14 o -0.09) | -0.12^**^ (-0.14 o -0.09) |
| **Previous vs. never smoker** | -0.06^**^ (-0.09 to -0.04) | -0.07^**^ (-0.09 to -0.04) | -0.06^**^ (-0.09 to -0.04) |
| **Current vs. never smoker** | -0.16^**^ (-0.20 to -0.12) | -0.16^**^ (-0.20 to -0.12) | -0.16^**^ (-0.20 to -0.12) |
| **TDI** | -0.02^**^ (-0.03 to -0.01) | -0.02^**^ (-0.03 to -0.01) | -0.02^**^ (-0.03 to -0.01) |
| **SBP, *mmHg*** | 0.02^**^ (0.01 to 0.03) | 0.02^**^ (0.01 to 0.03) | 0.02^**^ (0.01 to 0.03) |
| **DBP, *mmHg*** | -0.04^**^ (-0.05 to -0.04) | -0.04^**^ (-0.05 to -0.04) | -0.05^**^ (-0.05 to -0.04) |
| **BMI, *kg/m^2^*** | -0.05^**^ (-0.06 to -0.04) | -0.05^**^ (-0.06 to -0.04) | -0.05^**^ (-0.06 to -0.04) |
| **Metabolic Equivalent Task, *minutes weekly*** | 0.00 (0.00 to 0.01) | 0.00 (0.00 to 0.01) | 0.00 (0.00 to 0.01) |
| **Diabetes mellitus** | -0.27^**^ (-0.31 to -0.22) | -0.27^**^ (-0.31 to -0.22) | -0.27^**^ (-0.31 to -0.22) |
| **Non HDL cholesterol, *mmol/L*** | 0.02^**^ (-0.01 to 0.03) | 0.02^**^ (-0.01 to 0.03) | 0.02^**^ (-0.01 to 0.03) |
| **Estimated intracranial volume** | 0.230^**^ (0.28 to 0.31) | 0.30^**^ (0.28 to 0.31) | 0.30^**^ (0.28 to 0.31) |
| **Alcohol x age** | -0.01 (-0.02 to 8e-4) |  |  |
| **Alcohol x sex** |  | 0.00 (-0.01 to 0.01) |  |
| **Alcohol x Apoe 1** |  |  | 0.01 (-0.02 to 0.03) |
| **Alcohol x Apoe 2** |  |  | 0.05 (-0.01 to 0.12) |
| **Constant** | 0.10^**^ (0.05 to 0.14) | 0.10^**^ (0.06 to 0.14) | 0.10^**^ (0.06 to 0.15) |
| **Observations** | 22,007 | 22,007 | 22,007 |
| **R^2^** | 0.510 | 0.51 | 0.51 |
| **Adjusted R^2^** | 0.509 | 0.51 | 0.51 |
| **Residual Std. Error** | 0.70 (df = 21980) | 0.70 (df = 21980) | 0.70 (df = 21977) |
| **F Statistic** | 878.252^**^ (df = 26; 21980) | 878.029^**^ (df = 26; 21980) | 787.592^**^ (df = 29; 21977) |

**Table 16: Regression models for non-significant interactions with alcohol in predicting total grey matter volume (standardised estimates). Abbreviations: TDI – Townsend Deprivation Index, SBP – systolic blood pressure, DBP – diastolic blood pressure, BMI – body mass index, HDL – high-density lipoprotein. ^*^ uncorrected p < .05; ^**^ uncorrected p < .01.**

|  | **Quintile 2 drinkers** | **Quintile 3 drinkers** | **Quintile 4 drinkers** | **Quintile 5 drinkers** |
| --- | --- | --- | --- | --- |
|  | | | | |
| **Alcohol total weekly volume, *units*** | -0.02^*^(-0.03 0.00) | -0.02^*^(-0.03,0.00) | 0.01 (-0.01,0.01) | **-0.01^***^(-0.01,0.00)** |
| **<Monthly binge** | -0.02(-0.09, 0.04) | -0.02(-0.09, 0.04) | **-0.12^***^(-0.20, -0.05)** | -0.05(-0.15, 0.05) |
| **Monthly binge** | -0.01(-0.10, 0.08) | -0.01(-0.10, 0.08) | **-0.13^***^(-0.22, -0.05)** | -0.01(-0.12, 0.09) |
| **Weekly binge** | -0.01(-0.10, 0.08) | -0.01(-0.10, 0.08) | -0.07(-0.15, 0.01) | -0.05(-0.14, 0.04) |
| **Daily binge** | -0.23 -0.58, 0.12) | -0.23 (-0.58, 0.12) | -0.20^*^(-0.36, -0.04) | -0.19^**^(-0.30,-0.08) |
| **Age, *years*** | -0.10 (-0.65, 0.45) | -0.10 (-0.65, 0.45) | -0.17 (-0.71, 0.38) | 0.03 (-0.50, 0.55) |
| **Diabetes mellitus** | **-0.28^***^(-0.43, -0.13)** | **-0.28^***^(-0.43, -0.13)** | **-0.22^***^(-0.37, -0.08)** | **-0.30^***^ (-0.41, -0.18)** |
| **Sex** | -3.40^*^(-6.31, -0.50) | -3.40^*^(-6.31, -0.50) | -0.48(-3.37, 2.41) | -3.12(-6.35, 0.10) |
| **Age^2^** | 0.00(-0.01, 0.01) | 0.00(-0.01, 0.01) | 0.00(-0.01, 0.01) | 0.00(-0.01, 0.01) |
| **Age^3^** | 0.00 (0.00,0.00) | 0.00 (0.00,0.00) | 0.00 (0.00,0.00) | 0.00(-0.00,0.00) |
| **SBP, *mmHg*** | 0.00(0.00,0.00) | 0.00 (0.00,0.00) | 0.00 (0.00,0.00) | 0.00 (0.00,0.00) |
| **DBP, *mmHg*** | **-0.01^***^(-0.01,0.00)** | **-0.01^***^(-0.01,0.00)** | -0.00 (-0.01,0.00) | **-0.01^***^(-0.01,0.00)** |
| **TDI** | -0.01(-0.02,0.00) | -0.01(-0.02,0.00) | -0.01(-0.02,0.00) | -0.01(-0.02,0.00) |
| **BMI, *kg/m^2^*** | **-0.01^***^(-0.02,0.00)** | **-0.01^***^(-0.02,0.00)** | **-0.02^***^(-0.03, -0.01)** | **-0.01^***^ (-0.02,0.00)** |
| **Non-HDL cholesterol, *mmol/L*** | **0.05^***^(0.02, 0.07)** | **0.05^***^ (0.02, 0.07)** | 0.03 (0.00,0.05) | 0.01 (-0.02, 0.03) |
| **Site 1** | **-0.16^***^(-0.24, -0.09)** | **-0.16^***^(-0.24, -0.09)** | **-0.14^***^(-0.22, -0.07)** | **-0.13^***^(-0.21, -0.06)** |
| **Site 2** | **-0.13^***^(-0.19, -0.06)** | **-0.13^***^(-0.19, -0.06)** | **-0.11^***^(-0.17, -0.04)** | **-0.13^***^(-0.19, -0.07)** |
| **Metabolic Equivalent Task, *minutes weekly*** | 0.00 (0.00, 0.00) | 0.00 (0.00, 0.00) | 0.00 (0.00, 0.00) | 0.00 (0.00, 0.00) |
| **Estimated intracranial volume** | **2.42^***^ (2.13, 2.70)** | **2.42^***^ (2.13, 2.70)** | **2.55^***^ (2.26, 2.84)** | **2.99^***^ (2.71, 3.26)** |
| **Previous vs. never smoker** | **-0.12^***^(-0.18,-0.06)** | **-0.12^***^(-0.18,-0.06)** | -0.01(-0.06, 0.05) | -0.06^*^(-0.11,-0.003) |
| **Current vs. never smoker** | **-0.18^***^(-0.30, -0.06)** | **-0.18^***^(-0.30,-0.06)** | **-0.20^***^(-0.31, -0.09)** | -0.10^*^(-0.19,-0.02) |
| **Higher degree vs. no educational qualification** | 0.00(-0.14, 0.13) | 0.00 (-0.14, 0.13) | -0.07 (-0.21, 0.07) | -0.07 (-0.20, 0.05) |
| **A levels vs. no educational qualification** | 0.00(-0.15, 0.15) | 0.00(-0.15, 0.15) | -0.09 (-0.24, 0.07) | -0.07 (-0.21, 0.06) |
| **GCSEs vs. no educational qualification** | 0.02(-0.13, 0.16) | 0.02(-0.13, 0.16) | -0.06 (-0.21, 0.09) | -0.05 (-0.18, 0.08) |
| **CSEs vs. no educational qualification** | 0.09(-0.13, 0.30) | 0.09(-0.13, 0.30) | 0.09 (-0.11, 0.29) | -0.05 (-0.23, 0.12) |
| **NVQ vs. no educational educational qualification** | -0.04(-0.21, 0.14) | -0.04 (-0.21, 0.14) | -0.15 (-0.33, 0.02) | 0.00(-0.15, 0.15) |
| **Professional qualification vs. no educational qualification** | 0.10(-0.07, 0.28) | 0.10(-0.07, 0.28) | 0.01 (-0.17, 0.20) | 0.16 (-0.02, 0.33) |
| **Age x sex** | 0.10 (-0.01, 0.21) | 0.10 (-0.01, 0.21) | 0.00(-0.11, 0.11) | 0.11 (-0.01, 0.23) |
| **Age^2^ x sex** | 0.00 (0.00,0.00) | 0.00 (0.00,0.00) | 0.00 (0.00,0.00) | 0.00 (0.00, 0.00) |
| **Constant** | 2.38 (-7.39,12.16) | 2.38 (-7.39, 12.16) | 3.44 (-6.32, 13.19) | 0.01(-9.39, 9.41) |
| **N** | 2,879 | 2,879 | 2,898 | 2,911 |
| **R^2^** | 0.48 | 0.48 | 0.49 | 0.52 |
| **Adjusted R^2^** | 0.48 | 0.48 | 0.48 | 0.52 |
| **Residual Std. Error** | 0.72 (df = 2849) | 0.72 (df = 2849) | 0.72 (df = 2868) | 0.70 (df = 2881) |
| **F Statistic** | 91.62^**^ (df = 29; 2849) | 91.62^**^ (df = 29; 2849) | 94.08^**^ (df = 29; 2868) | 108.18^**^ (df = 29; 2881) |
|  | | | | |

**Table 17: Regression models examining binging frequency in predicting total grey matter volume, independently of total alcohol intake consumed (standardised estimates). Abbreviations: TDI – Townsend Deprivation Index, SBP – systolic blood pressure, DBP – diastolic blood pressure, BMI – body mass index, HDL – high-density lipoprotein. ^*^ uncorrected p < .05; ^***^Bonferroni threshold p=0.012.**

***

***

**Figure 22: Associations between weekly units of alcohol intake and total grey matter volume for each alcoholic beverage type consumed. Subjects included are those solely drink one alcoholic beverage type. Coloured bars show regression coefficients (estimates) and 95% CI separately for wine (n=5080), beer (n=1193) and spirits (n=329) drinkers. Models were adjusted for: age, sex, age*sex, age^2^, age^3^, age^2^*sex, educational qualifications, blood pressure, diabetes mellitus, smoking status, Townsend Deprivation Index, body mass index, cholesterol, high-density lipoprotein, imaging site, estimated intracranial volume and Metabolic Equivalent Task minutes weekly.**

**
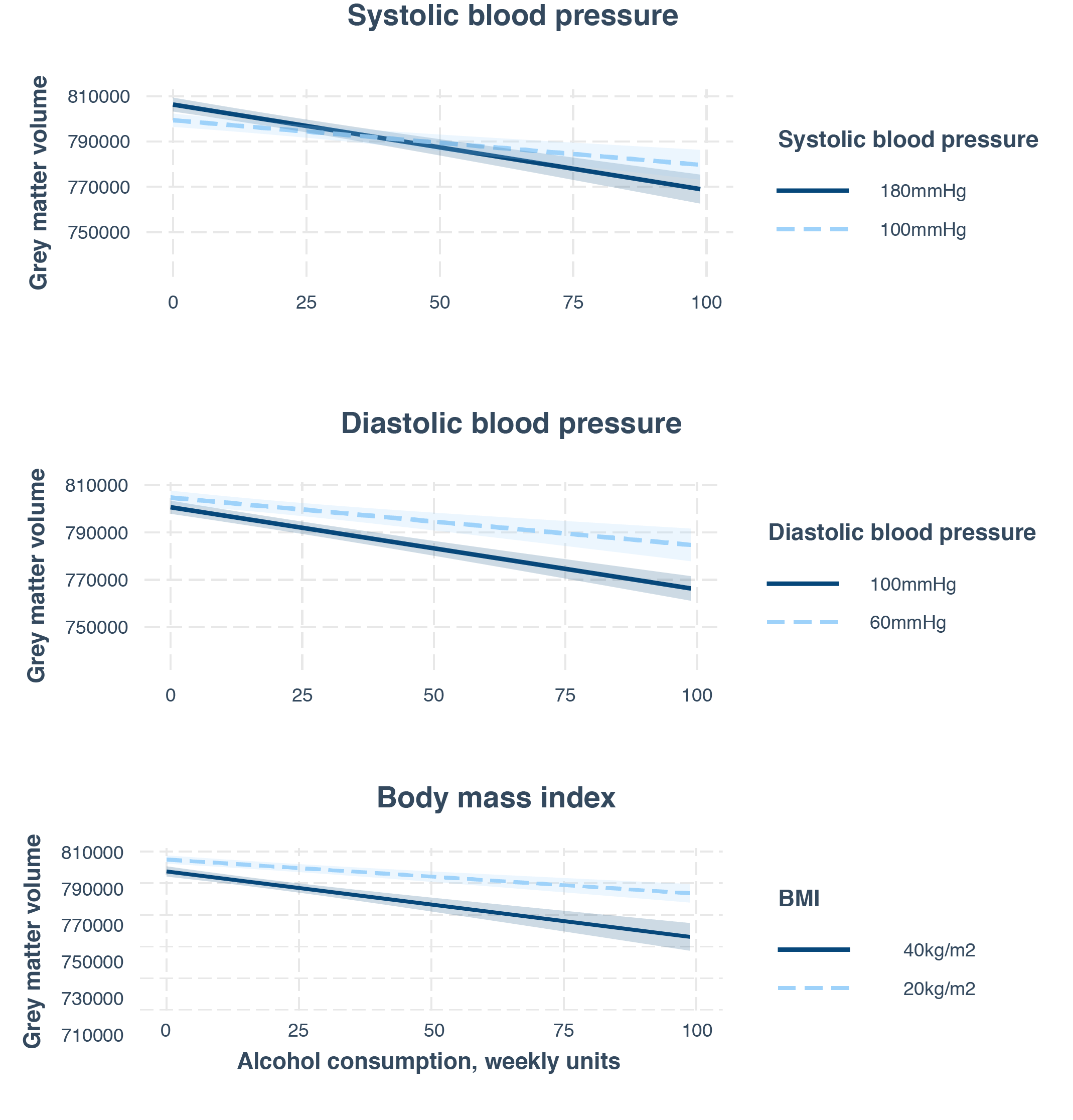
**

**Figure 23: Predicted change in total grey matter volume according to alcohol intake at different blood pressure and body mass index levels, when all other confounders are held constant. Predictions generated based on regression models adjusted for: age, age^2^, age^3,^ sex, age x sex, age^2^ x sex, diabetes mellitus, blood pressure, body mass index, total cholesterol, high-density lipoprotein, imaging site, Metabolic Equivalent Task minutes weekly, smoking status, educational qualifications, Townsend Deprivation Index, estimated intracranial size. 95% confidence intervals are shaded.**

1. Raichle ME. The brain's default mode network. Annual review of neuroscience. 2015;38:433-47.

2. Damoiseaux JS, Rombouts S, Barkhof F, Scheltens P, Stam CJ, Smith SM, et al. Consistent resting-state networks across healthy subjects. Proceedings of the national academy of sciences. 2006;103(37):13848-53.

3. Smitha K, Akhil Raja K, Arun K, Rajesh P, Thomas B, Kapilamoorthy T, et al. Resting state fMRI: A review on methods in resting state connectivity analysis and resting state networks. The neuroradiology journal. 2017;30(4):305-17.
